# A cross country comparison for the burden of cardiovascular disease attributable to tobacco exposure in China, Japan, USA and world

**DOI:** 10.1186/s12889-020-09031-7

**Published:** 2020-06-08

**Authors:** Xiaomei Wu, Bo Zhu, Shuang Xu, Yifei Bi, Yong Liu, Jingpu Shi

**Affiliations:** 1 Department of Clinical Epidemiology and Center of Evidence Based Medicine, Institute of Cardiovascular Diseases, The First Hospital of China Medical University, No.155 Nanjing Bei Street, Heping District, Shenyang, 110001 LiaoningProvince China; 2 Department of Cancer Prevention and Treatment, Cancer Hospital of China Medical University/Liaoning Cancer Hospital & Institute, Shenyang, China; 3 Library of China Medical University, Shenyang, China; 4 Department of Psychology, University of York, York, UK; 5 Periodontology and Preventive Dentistry, Saarland University, Saarbrücken, Germany

**Keywords:** Tobacco exposure, Cardiovascular disease, Age-period-cohort analysis

## Abstract

**Background:**

Tobacco exposure (TE) is the major contributor for CVD mortality, but few published studies on CVD mortality attributable to TE have analyzed the potential reasons underlying long-term trends in China. Our studysought to find the potential reasons and compared CVD mortality attributable to TE in China, Japan, the United States of America (USA), and the world between 1990 and 2017.

**Methods:**

The mortality data in China, Japan, the USA, and the world were obtained from Global Burden of Disease Study 2017(GBD 2017). Joinpoint regression was used to assess the trend magnitude and directions over time for CVD mortality, while the age-period-cohort method was used to analyzethe temporal trends of CVD mortality according to age, period, and cohort.

**Results:**

A significant downward trend was found in the age-standardised mortality rate (ASMR) of CVD attributable to smoking in four regions. China had the smallest decline and the Chinese ASMR became the highest in 2017. All the annual net drifts in the four regions were negative and the local drifts were below zero. The longitudinal age curves of CVD mortality attributable to smoking increased in four regions,with China having the largest increase. The period or cohort RRs indicated a decline, and China had the smallest decline. The researchers further analyzed the IHD and stroke trends, finding that the morality and period or cohort RR of IHD in China was always at a high level.

**Conclusions:**

CVD mortality attributable to TE declined in four regions, and was highest in China. The proportion of IHD mortality attributable to TE was similar to stroke, which significantly changed the traditional cognition of CVD composition, but the control measure was not sufficient for IHD in China.

## Background

Accompanying economic development and urbanisation, disease patterns have shifted from infectious to noncommunicable diseases. As a noncommunicable disease, cardiovascular disease (CVD) is the world’s leading cause of premature morbidity and mortality. An estimated 17.8 million people died of CVD in 2017, representing the cause of 30% of total deaths from around the world [1]. In China, CVD mortality increased from 187.58 per 100,000 people in 1990 to 309.95 per 100,000 people in 2017, resulting in a very high CVD burden. The estimated number of CVD caused deaths almost doubled from 2.25 million in 1990 to 4.38 million in 2017 [2]. China has the world’s highest burden of CVD and faces enormous challenges in tackling the burden of CVD [3].

Tobacco exposure (TE) mainly includes active smoking and second-hand smoke (SHS). Globally, almost 7 million people died from TE, includingapproximately 6 million tobacco users and an estimated 890,000 among non-smokers exposed to SHS [4]. On average, long-term smokerslost at least 10 years of life. Furthermore, over 22,000 people die from TE each day, equivalent to one person dying every 4 s. Smoking is widely recognised as a risk factor for premature morbidity and mortality, and it has been challenging to achieve adequate monitoring of smoking levels and trends throughout the world. Nearly two-thirds of the world’s smokers live in ten countries, with China accounting for 30% of them. In 2017, active smoking became the primary leading risk factor for DALYs (disability adjusted life years) and the second risk factor for death in China [5].

TE causes CVD through atherosclerosis and thrombosis. The mechanism of smoking-induced atherosclerosis and thrombosisinvolves oxidative stress mediated by cigarette smoke or endogenous oxygen free radicals, which has multiple thrombosis-promoting and anti-fibrinolysis effects,leading to intravascular thrombosis and the triggering of acute cardiovascular events [6–8]. Eventually,TE can result in plaque and blood clots, blood flow restriction, as well as heart attacks and strokes. Similar to heart disease, strokes have a high risk of death and stroke survivors may experience disastrous disabling conditions, including paralysis or the loss of vision or speech [9–11]. Several epidemiological studies have concluded that smoking is a major cause of CVD and is the cause of a quarter of deaths from CVD [12]. SHS also can cause CVD, including heart attacks and strokes. Non-smokers who breathe SHS at home or work increase their risk of developing heart disease by 25–30%,while SHS increases the risk for stroke by 20–30% [13]. The World Health Organization (WHO) considered that TE is an entirely avoidable CVD risk factor [14]. The probability of TE varies according to age and gender, while different regions have different tobacco control strategies. The disease burden attributable to TE therefore varies according to age, sex, and region [15].

Although previous studies have analyzed disease caused by tobacco exposure, there has been a lack of analysis on the impact of traditional factors (such as age, period, and generation), and previous studies have been unable to find a suitable way of reducing harm from tobacco exposure [16, 17]. In the present study, the researchers wanted to find the potential reasons underlying the long-term trends and the differences betweenage-groups. Furthermore, this study compares CVD mortality attributable to TE between China, Japan, the United States of America (USA), and the world in the period 1990 to 2017. The world represents the average level, the USA was among the first countries to implement tobacco control measures, while Japan is a neighbouring country to China and its population is of a similar race and harm level from smoking for the population. By comparing China with the aforementioned regions, it was hoped that methods could be found to reduce CVD mortality attributable to TE in China.

## Methods

### Data source

Data was taken from the freely available GBD Data Tool repository,accessible from http://ghdx.healthdata.org/gbd-results-tool. To analyze the status of CVD mortality attributable to TE in China, the researchers extracted relevant data on CVD mortality and compared this with data from Japan, the USA, and the world. In 2017, ischemic heart disease (IHD) and stroke were the top two causes of death in China [2]. This prompted the researchers to analyzethe changes of CVD attributable to TE using both the temporal trend and age-period-cohort methods, as well as to analyzethe changes of IHD and stroke to systematically and comprehensively reflect the impact of TE on CVD. CVD, IHD, and stroke were diagnosed according to the definitions from WHO clinical criteria and ICD 9.

### Statistical analysis

The age-standardized mortality rate (ASMR) was used to estimate the trend. Because the Chinese and American populations have different age structures, it is necessary to perform standardisation when comparing mortality trends. The ASMR (per 100,000 population) was calculated using the direct method, which is the sum of age-specific rates (*a*_*i*_, where *i* denotes the *i*^*th*^ age class) and the number of persons (or weight) (*w*_*i*_) in the same age subgroup *i* of the chosen reference standard population, divided by the sum of standard population weights:

$$ \mathrm{ASR}=\frac{\sum_{i=1}^A{a}_i{w}_i}{\sum \limits_{i=1}^A{w}_i}\times \mathrm{100,000} $$

In order to assess the magnitude and direction of mortality rate trends over time, Joinpoint software (Version 4.7.0.0) was used to calculate the average annual percentage change (AAPC) and thecorresponding 95% CIs by Joinpoint regressionanalysis from the 1990–2017 data.

The age-period-cohort (APC) model is popularly used to estimate net age, period, and cohort effects on disease incidence and mortality. The estimable parameters of the APC analysis were longitudinal age-specific rates, period, and cohort rate ratios, as well as local drifts with net drift. The longitudinal age curve indicates the fitted longitudinal age-specific rates in reference cohorts adjusted for period deviations, while the period (or cohort) RR is the period (or cohort) relative risk adjusted for age and nonlinear cohort (or period) effects in a period (or cohort) versus the reference one. Net drift is the overall log-linear trend by calendar period and birth cohort, and indicates the overall annual percentage change, while local drifts is the log-linear trend by calendar period and birth cohort for each age group to indicate annual percentage changes for each age group [18–20].

The present study used ACP to analyze the trend of CVD mortality attributable to TE under the effects of age, period, and cohort. The age effect is concerned with age-related physiological and pathological changes which affect disease mortality rates. The period effect is related to disease mortality rate changes caused by various events over time, such as effective treatments and the implementation of screening. Finally, cohort effects reflect disease mortality rate differences between generations as a consequence of lifestyle over time or different exposure risk factors.

The general logarithmic linear form of the APC model is: ρ = *α*_*a*_ + *π*_*p*_ + *γ*_*c*_. It can then be transformed into the form of age-period: ${\rho}_{pa}=\mu +\left({\alpha}_L-{\gamma}_L\right)\left(\alpha -\overline{\alpha}\right)+\left({\pi}_L-{\gamma}_L\right)\left(p-\overline{p}\right)+\overset{\sim }{\alpha_a}+\overset{\sim }{\pi_p}+\overset{\sim }{\gamma_c}$, or the form of age-cohort: ${\rho}_{pa}=\mu +\left({\alpha}_L+{\gamma}_L\right)\left(\alpha -\overline{\alpha}\right)+\left({\pi}_L+{\gamma}_L\right)\left(c-\overline{c}\right)+\overset{\sim }{\alpha_a}+\overset{\sim }{\pi_p}+\overset{\sim }{\gamma_c}$. $\overset{\sim }{\alpha_a}$, $\overset{\sim }{\pi_p}$ and $\overset{\sim }{\gamma_c}$ represent age, age, and cohort deviation, respectively. *π*_*L*_ + *γ*_*L*_ represents net drift. The longitudinal age curve represents fitted longitudinal age-specific rates in reference to cohorts adjusted for period deviations. Period rate ratios (period RRs) represent the ratio of age-specific rates in a period relative to the reference period. Cohort rate ratios (cohort RRs) represents the ratio of age-specific rates in a cohort relative to the reference cohort. In the APC model, it was necessary for the collected data to be converted to successive 5-yearage groups and consecutive 5-year periods. Because the GBD dataset did not provide successive 5-yearage groups for those under 29 or over 80 years of age related to smoking, the CVD mortality rates attributable to smoking were recoded into successive 5-yearage groups (30–34 years to 75–79 years) and consecutive 5-year periods (1990–1994 to 2015–2017). Since the GBD dataset did not provide successive 5-yearage groupsunder the age of 25 or over 80 for SHS, the CVD mortality rates attributable to SHS were recoded into successive 5-yearage groups (25–29 years to 75–79 years) and consecutive 5-year periods (1990–1994 to 2015–2017). The “apc” packages were used to perform APCM in R statistical software (R version 3.5.1), and *p* < 0.05 was considered significant.

## Results

### (1) The temporal trend in the age-standardized mortality rate (ASMR) of CVD (including IHD and stroke) attributable to TE from 1990 to 2017

#### Smoking

**Fig. 1:**
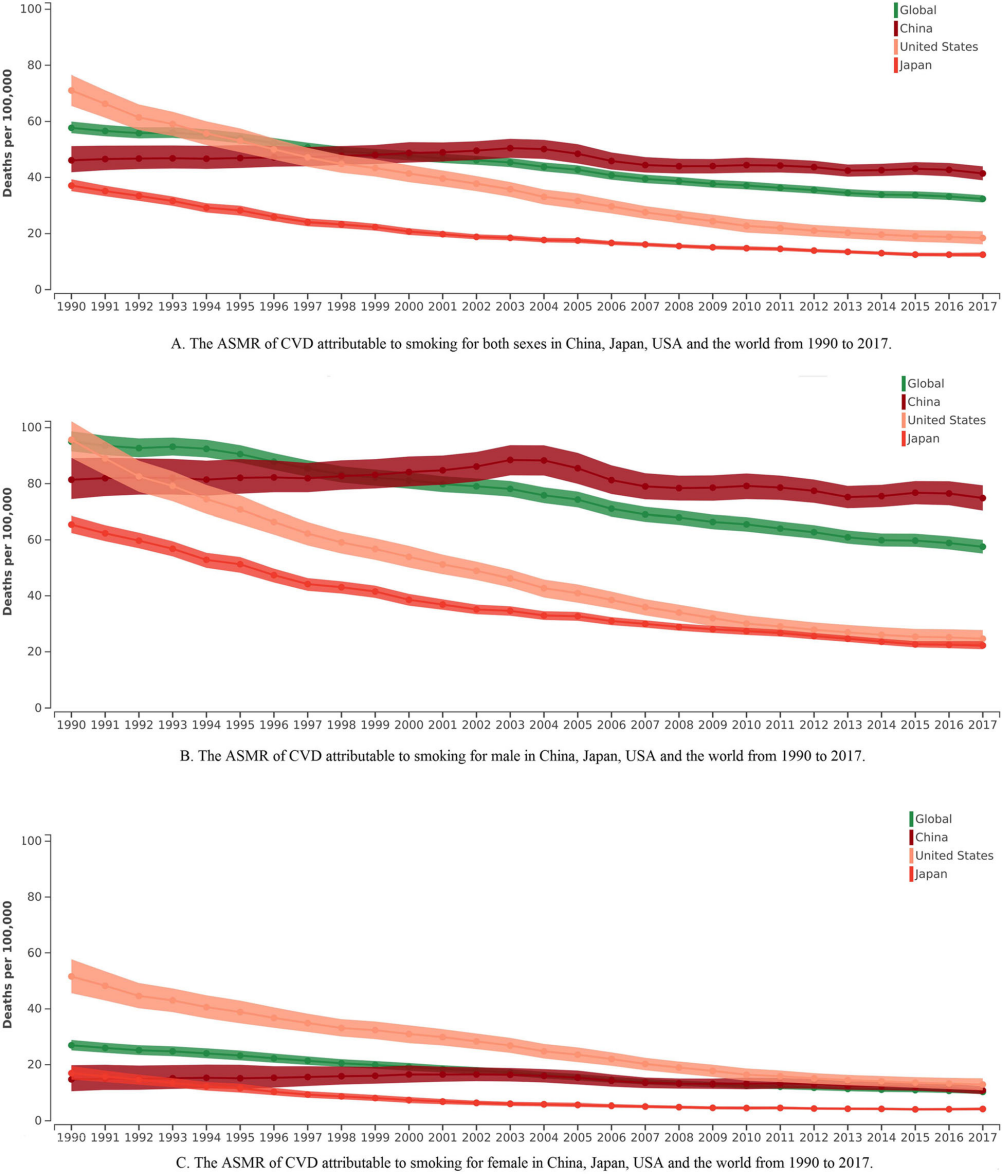
The ASMR of CVD attributable to smoking in China, Japan, the USA, and the world from 1990 to 2017

For both sexes, the ASMR of CVD attributable to smoking in China, Japan, the USA, and the world significantly decreased by 0.4% (95%CI: 0.0–0.7%), 4.0% (95%CI: 3.7–4.3%), 4.9% (95%CI: 4.7–5.1%), and 2.1% (95%CI: 1.8–2.4%) per year, respectively (Fig. 1). The ASMR of IHD attributable to smoking in Japan, the USA, and the world significantly decreased by 4.2% (95%CI: 3.9–4.5%), 5.1% (95%CI: 4.9–5.3%), and 2.2% (95%CI: 2.0–2.4%) per year. Nonetheless in China, the ASMR of IHD attributable to smoking in China increased by 0.6% (95%CI: 0.1–1.0%) (Figure S1). There was a significant downward trend in the ASMR of stroke attributable to smoking in China, Japan, the USA, and the world. In 1990, China was not the first-ranked country for this measure, but it was in 2017 (Figure S2). The CVD, IHD, and stroke attributable to smoking rates was similar between males and females in each region. The above detailed results are shown in Table 1.

**Table 1:** The temporal trend of the mortality rate of CVD, IHD, and stroke attributable to smoking in China, Japan, the USA, and the world from 1990 to 2017

		China		Japan		USA		World	
		AAPC (%)	95%CI (%)	AAPC (%)	95%CI (%)	AAPC (%)	95%CI (%)	AAPC (%)	95%CI (%)
**CVD**	both sexes	−0.4*	(− 0.7,0.0)	− 4.0*	(− 4.3,-3.7)	− 4.9*	(− 5.1,-4.7)	− 2.1*	(− 2.4,-1.8)
	male	−0.3	(− 0.6,0.0)	− 3.9*	(− 4.4,-3.5)	− 4.9*	(− 5.1,-4.7)	− 1.8*	(− 2.2,-1.5)
	female	− 1.1*	(− 1.4,-0.7)	−5.1*	(− 5.4,-4.8)	− 5.0*	(− 5.2,-4.8)	− 3.5*	(− 3.6,-3.4)
**IHD**	both sexes	0.6*	(0.1,1.0)	− 4.2*	(− 4.5,-3.9)	−5.1*	(− 5.3,-4.9)	− 2.2*	(− 2.4,-2.0)
	male	0.7*	(0.3,1.0)	− 4.1*	(− 4.5,-3.7)	−5.1*	(− 5.3,-4.9)	−1.9*	(− 2.2,-1.7)
	female	0.2	(− 0.4,0.8)	−5.4*	(−5.8,-5.1)	− 5.2*	(− 5.4,-5.0)	− 3.6*	(− 3.7,-3.4)
**Stroke**	both sexes	−1.2*	(− 1.4,0.9)	− 4.5*	(− 4.7,-4.2)	− 4.3*	(− 4.6,-4.1)	− 2.0*	(− 2.2,-1.8)
	male	− 1.0*	(− 1.2,-0.8)	− 4.5*	(− 4.9,− 4.1)	-4.1*	(− 4.3,-3.9)	−1.7*	(− 1.9,-1.5)
	female	− 2.3*	(− 2.9,-1.8)	− 5.4*	(− 5.8,-5.0)	− 4.7*	(− 4.9,-4.4)	− 3.5*	(− 3.6,-3.3)

*: statistically significant (*p* < 0.05); AAPC: average annual percent change

#### Second-hand smoke

For both sexes, the ASMR of CVD attributable to SHS in China, Japan, the USA, and the world significantly decreased by 1.2% (95%CI: 0.7–1.7%), 3.8% (95%CI: 3.5–4.1%), 3.9% (95%CI: 3.6–4.2%), and 1.8% (95%CI: 1.5–2.0%) per year, respectively (Figure S3). The ASMR of IHD attributable to SHS in Japan, the USA, and the world significantly decreased by 3.8% (95%CI: 3.5–4.0%), 4.1% (95%CI: 3.8–4.4%), and 1.6% (95%CI: 1.3–1.9%) per year, but there was no significant trend change in China (Figure S4). There were therefore significant downward trends in the ASMR of stroke attributable to SHS in China, Japan, the USA, and the world (Figure S5). The change of CVD, IHD, and stroke attributable to SHS in males and females was similar within each region. The results described above are shown in Table S1.

### (2) APC analysis of the CVD mortality rate (including IHD and stroke) attributable to TE from 1990 to 2017

**Fig. 2:**
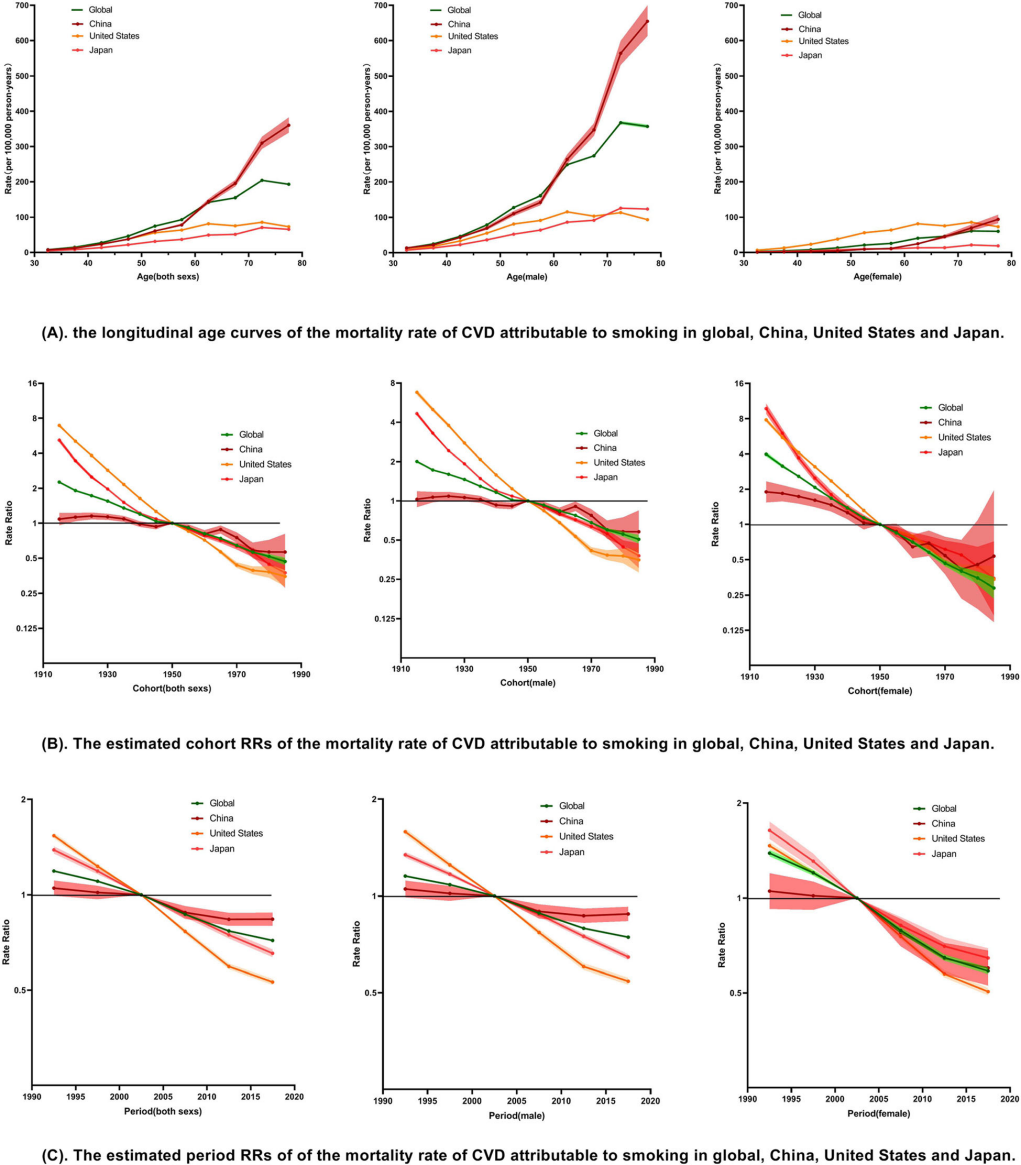
The APC results of CVD attributable to smoking in China, Japan, the USA, and the world from 1990 to 2017

**Smoking:** For both sexes in the same birth cohort in China,the mortality rate of CVD attributable to smoking rapidly increased from 7.38 (95%CI: 6.34, 8.58) per 100,000 in the 30–34 age-group to 360.15 (95%CI: 338.97, 382.67) per 100,000 in the 75–79 age-group in China. Similar changes are observed in Japan, the USA, and the world (Fig. 2a). The mortality rate of IHD and stroke attributable to smoking also increased between the 30–34 age-group and the 75–79 age-group (Figures S6A and S7A). All the period RRs of CVD and stroke showed a decreasing trend from 1990 to 2017 in the four regions studied (Figs. 2b and S7B). The period RRs of IHD in Japan, the USA, and the world also showed a downward trend from 1990 to 2017. In China, the period RRs of IHD originally showed a downward trend, but then showed an upward trend (Figure S6B). All the cohort RRs of CVD and stroke showed decreasing trends in the four regions (Fig. 2c and S7C). The cohort RRs of IHD in Japan, the USA, and the world also showed a downward trend from 1990 to 2017, but in China the cohort RRs of IHD showed an upward trend, and then showed a downward trend (Figure S6C). The effects of age, period, and cohort on CVD, IHD, and stroke attributable to smoking were similar for males in females in each region.

For CVD, the overall annual net drifts in China, Japan, the USA, and the world werebelow zero. For IHD, the overall annual net drifts in Japan, the USA, and the world were below zero, but in China this was 0.01% (95%CI: − 0.22, 0.23%). For stroke, the overall annual net drifts in China, Japan, the USA, and the world werebelow zero. All the local drift values increased by age groupsin China, decreased in Japan and the USA, and fluctuated in the world (Figure S8). The changes to CVD, IHD, and stroke were similar for males and females within each region. The above detailed results are shown in Table 2.

**Table 2:** The net drift value of the mortality rate of CVD, IHD and stroke attributable to smoking

		China		Japan		USA		World	
		Net Drift (%/year)	95%CI	Net Drift (%/year)	95%CI	Net Drift (%/year)	95%CI	Net Drift (%/year)	95%CI
**CVD**	both sexes	−1.049	(−0.837,-1.26)	− 2.983	(− 2.855,-3.11)	− 4.341	(− 4.25,-4.433)	−2.126	(− 2.085,-2.167)
	male	− 0.847	(− 0.624,-1.071)	− 2.895	(− 2.8,-2.989)	− 4.359	(− 4.256,-4.462)	−1.845	(− 1.803,-1.888)
	female	−2.507	(−1.817,-3.192)	−3.774	(− 3.458,-4.089)	− 4.379	(− 4.259,-4.499)	−3.593	(− 3.488,-3.698)
**IHD**	both sexes	0.005	(0.234,-0.224)	−2.982	(− 2.835,-3.128)	− 4.472	(− 4.369,-4.576)	− 2.100	(− 2.058,-2.143)
	male	0.198	(0.434,-0.037)	− 2.890	(− 2.771,-3.008)	−4.564	(− 4.451,-4.676)	−1.838	(− 1.798,-1.878)
	female	−1.323	(− 0.709,-1.932)	−3.799	(− 3.447,-4.149)	− 4.319	(− 4.173,-4.465)	− 3.420	(−3.315,-3.526)
**Stroke**	both sexes	− 1.904	(− 1.688,-2.12)	−3.665	(− 3.521,-3.809)	− 4.100	(− 3.996,-4.203)	−2.236	(− 2.165,-2.307)
	male	− 1.684	(−1.45,-1.917)	− 3.594	(− 3.445,-3.744)	−3.631	(− 3.492,-3.771)	− 1.901	(− 1.823,-1.979)
	female	− 3.618	(−2.834,-4.395)	−4.327	(− 4.007,-4.646)	− 4.688	(− 4.533,-4.842)	−4.024	(−3.88,-4.167)

#### Second-hand smoke

All the results for second-hand smoke were similar to those of smokers for each disease and within each region. The results are shown in Table S2.

## Discussion

Globally, a third of deaths can be attributed to CVD, while TE is the majorcontributor and causes approximately 3 million cardiovascular deaths per year [21]. In 2019, WHO reported estimates for the prevalence of smoking in all countries in response to the global tobacco epidemic [22]. From the report, it is found that China has the highest smoking prevalence compared to the other three regions in this study. Meanwhile, the smoking prevalence decline was smallest in China and the country has the highest level of exposure to second-hand smoke. Due to the possibility of TE, sex is an important demographic risk factor for CVD [23]. From the results in this study, it is found that the mortality rates of CVD, IHD, and stroke attributable to smoking and SHS were similar for both sexes. It is suggested that males were the primary victims from smoking, while women were the primary victims from SHS.

The results show a significant downward trend in ASMRs for CVD attributable to smoking from 1990 to 2017 in the four regions studied. Compared to the world, the ASMRs of CVD, IHD and stroke attributable to smoking had the biggest decline in the USA and Japan,while China had the smallest rate of decline from 1990 to 2017. It is found that in China, the ASMRs of CVD and IHD attributable to smoking became the highest in 2017, while the ASMR of stroke continued to be highest in China from 1990 to 2017. All the ASMRs of CVD, IHD, and stroke attributable to smoking in China were higher compared to the world, and the ASMRs of CVD, IHD, and stroke attributable to smoking in Japan and the USA were lower than those in the world. For SHS in China, the ratio of SHS to smoking in the ASMR of CVD ranged from one-quarter to one-third in the period 1990 to 2017. Compared with the other three regions, SHS exposure had caused a serious CVD burden in China. One study on SHS found that non-smoking women married to husbands that smoke had an almost 40% increased mortality risk,while the incidence of heart disease also increased [18]. A national population-based case-control study found that SHS can increase the risk of death by 10% for all forms of strokes by adjusting for related variables [19]. In 2017, the researchers also found all the ASMRs of CVD, IHD, and stroke attributable to SHS in China were higher than those in the world, while the results for Japan and USA were the reverse.

In 2017, the researchers found that China had the highest levels of ASMRs of CVD, IHD, and stroke attributable to TE. This may be due to when tobacco control measures were implemented. Tobacco control is a global health priority for non-communicable diseases. The USA and Japan started tobacco control relatively early compared to the world, while this only happened later in China [22]. The WHO Framework Convention on Tobacco Control (FCTC) was formalised global commitment by 180 countries and began to be implemented in 2005 [24]. The United States Congress passed the Federal Cigarette Labelling and Advertising Act and the Cigarette Act in 1965 and 1969, respectively. The Family Smoking Prevention and Tobacco Control Act, which passed in 2007, was stricter than FCTC [25]. In Japan, tobacco control started later than most developed countries. In 1978, a non-governmental movement for tobacco control was launched, and in 1985 the tobacco monopoly system was reformed [25]. In 2010, tax on cigaretteswas raised by a large amount. China signed FCTC, which came into operation in 2006. China implemented restrictions on the packaging, advertising, and ingredients of tobacco products, for instance one cigarette with 15 mg of tar would be regarded as an unqualified products and not be allowed to enter the cigarette market [26].

In APC analysis, the longitudinal age curve reflects the impact of age on the mortality rate of disease. In this study, the longitudinal age curves of the mortality rate of CVD, IHD, and stroke attributable to smoking increased by age in China, Japan, the USA, and the world, with this increasing in China more than the other regions studied. Before age 60, the morality rates of CVD, IHD, and strokeattributable to smoking were at lower levels with slight differences in the four regions, but they became wider after the age of 60. In each age group, the morality rates of CVD, IHD, and strokeattributable to smoking were lowest in Japan. Similar results were also found for the mortality rate of CVD, IHD, and stroke attributable to SHS. Age is an important factor for CVD, and the results in the present study could be due to two reasons: First, long-term TE results in vascular damage; second, physical resistance declines with age [27]. In China, it is suggested that the elderly should be listed as key persons in the prevention and treatment of CVD, basic public health services should be optimised for them, and the screening and management of the elderly should be performed well.

In four regions, the period RRs indicate a downwards trend in the mortality rate of CVD attributable to smoking from 1990 to 2017. Similarly, the cohort RRs also indicate a downwards trend in CVD mortality from births in 1910 to births in 1990. China is found to have experienced the smallest decline in the cohort and period RRs, while the cohort and period RRs in femalesdeclined faster than for males. The changes of the period and cohort RRs for stroke were similar to CVD, while IHD was found to be little different from CVD. While the period and cohort RRs indicate a down trend in the USA, Japan, and the world, these fluctuated in China. In the four regions, all the net drifts were significant and below zero, with the exception of the net drift of IHD in China. All the SHS results are similar to those for smokers, while the results for males and females are also similar. Compared with the USA, there were significant differences in the morality pattern of CVD in China, since the proportion of stroke was higher, the proportion of IHD was lower, and the morality pattern of CVD in China was similar to Japan [28]. Similar patterns are also identifiable in Tables S3 and S4. Due to improvements to medical conditions in China, increased public health funding,and the early diagnosis and treatment of noncommunicable diseases such as CVD and cancer, the occurrence and death of CVD has been effectively controlled. In particular, there have been significant improvements in stroke mortality, which could be due to more attention paid to strokes due to its prevalence. But the results reflect that the downwards trend in the mortality rate of IHD is not obvious. Through comparison, it is found that the mortality rate of IHD decreased in the USA, where IHD is the main component of CVD. Meanwhile, the mortality rate of IHD also decreased in Japanwith a similar CVD composition to that of China in which IHD was not the main component of CVD. Based on China’s actual situation, China should learn from these two countries to explore appropriate measures for CVD and tobacco control.

A number of limitations are identified in the present study. First, GBD 2017 underwent many modifications and adjustmentsto its data sources, collation, and evaluation methods to fulfil missing data and improve its data quality and comparability, but it is still difficult to avoid bias. Therefore, the integrity and accuracy of the data in the present study was affected. Yet using the GBD database to show the long-term trends minimises the adverse effects,meaning that the bias from the GBD database will not have an intolerable impact to the present study andbarely affects the changes to the long-term trends [29–31]. Second, the APC model only regards population as the unit of observation and analysis, which could lead to ecological fallacy. Therefore, the results of this study must be further confirmed by future research. Third, the researchers only estimated the effects of age, period, and cohort on the CVD mortality data attributable to tobacco exposure, while no further analysis was conducted on other risk factors.

## Conclusions

In China, with its economic development and social progress, people have a certain level of understanding of the dangers of TE, resulting in a lower smoking prevalence and exposure to SHS in public areas, thereby leading to a decline in the mortality rate of CVD attributable to TE. In our study, we analyzed the long-term trends of CVD mortality attributable to TE under the effects of age, period, and cohort, and compared CVD burden in China, Japan, USA and the world. It is found that in four regions, CVD mortality attributable to TE had the smallest rate of decline in China from 1990 to 2017, and China had the highest levels of ASMRs of CVD, IHD, and stroke attributable to TE in 2017. It is also found that the mortality rate of CVD in older people was higher in China, probably because tobacco control happened relatively late in China. In addition, due to the changes of the traditional cognition of CVD composition in China, the proportion of IHD mortality attributable to TE was similar to stroke, but the control measures were not sufficient for IHD. Therefore, China should strengthen tobacco control measures for the key population (e.g., older people) and disease (e.g., IHD) to reduce CVD burden in the next few years.

## Supplementary information

**Additional file 1: Table S1.** The temporal trend in mortality rate of CVD, IHD and stroke attributable to secondhand smoke in China, Japan, USA and the world from 1990 to 2017. **Table S2.** The net drift value of the mortality rate of CVD, IHD and stroke attributable to secondhand smoke. **Table S3.** The percent of IHD and stroke in CVD attributable to smoking in China, Japan, USA and the world from 1990 to 2017. **Table S4.** The percent of IHD and stroke in CVD attributable to secondhand smoke in China, Japan, USA and the world from 1990 to 2017.

**Additional file 2: Figure S1.** The ASMR of IHD attributable to smoking in China, Japan, USA and the world from 1990 to 2017. **Figure S2.** The ASMR of stroke attributable to smoking in China, Japan, USA and the world from 1990 to 2017. **Figure S3.** The ASMR of CVD attributable to secondhand smoke in China, Japan, USA and the world from 1990 to 2017. **Figure S4.** The ASMR of IHD attributable to secondhand smoke in China, Japan, USA and the world from 1990 to 2017. **Figure S5.** The ASMR of stroke attributable to secondhand smoke in China, Japan, USA and the world from 1990 to 2017. **Figure S6.** The APC results of IHD attributable to smoking in China, Japan, USA and the world from 1990 to 2017. **Figure S7.** The APC results of stroke attributable to smoking in China, Japan, USA and the world from 1990 to 2017. **Figure S8.** The local drift with net drift values of the mortality rate of CVD, IHD and stroke attributable to smoking. **Figure S9.** The APC results of CVD attributable to secondhand smoke in China, Japan, USA and the world from 1990 to 2017. **Figure S10.** The APC results of IHD attributable to secondhand smoke in China, Japan, USA and the world from 1990 to 2017. **Figure S11.** The APC results of stroke attributable to secondhand smoke in China, Japan, USA and the world from 1990 to 2017. **Figure S12.** The local drift with net drift values of the mortality rate of CVD, IHD and stroke attributable to secondhand smoke.

## Data Availability

All our research data are obtained from GBD 2017, the website was http://ghdx.healthdata.org/gbd-results-tool.

## Abbreviations

CVD: Cardiovascular Disease
SHS: Secondhand smoke
WHO: World Health Organization
IHD: Ischemic heart disease
AAPC: Average annual percentage change
APC: The age–period–cohort
ASMR: Age-standardized mortality rate
