# Supplementary material for: A cross country comparison for the burden of cardiovascular disease attributable to tobacco exposure in China, Japan, USA and world
Source: BMC Public Health. 2020 Jun 8;20:888. doi: 10.1186/s12889-020-09031-7 (PMC7282071; doi:10.1186/s12889-020-09031-7)
Supplement: Supplementary file 1 — Additional file 1: Table S1. The temporal trend in mortality rate of CVD, IHD and stroke attributable to secondhand smoke in China, Japan, USA and the world from 1990 to 2017. Table S2. The net drift value of the mortality rate of CVD, IHD and stroke attributable to secondhand smoke. Table S3. The percent of IHD and stroke in CVD attributable to smoking in China, Japan, USA and the world from 1990 to 2017. Table S4. The percent of IHD and stroke in CVD attributable to secondhand smoke in China, Japan, USA and the world from 1990 to 2017. [file 12889_2020_9031_MOESM1_ESM.docx]

Table S1. The temporal trend in mortality rate of CVD, IHD and stroke attributable to secondhand smoke in China, Japan, USA and the world from 1990 to 2017.

|  |  | **China** |  | **Japan** |  | **USA** |  | **World** |  |
| --- | --- | --- | --- | --- | --- | --- | --- | --- | --- |
|  |  | **AAPC (%)** | **95%CI (%)** | **AAPC (%)** | **95%CI (%)** | **AAPC (%)** | **95%CI (%)** | **AAPC (%)** | **95%CI (%)** |
| **CVD** | both sexes | -1.2* | (-1.7,-0.7) | -3.8* | (-4.1,-3.5) | -3.9* | (-4.2,-3.6) | -1.8* | (-2.0,-1.5) |
|  | male | -0.6 | (-1.3,0.2) | -2.7* | (-3.1,-2.3) | -4.1* | (-4.4,-3.8) | -1.5* | (-1.8,-1.3) |
|  | female | -1.6* | (-2.1,-1.0) | -4.8* | (-5.1,-4.4) | -3.5* | (-3.8,-3.3) | -1.9* | (-2.1,-1.8) |
| **IHD** | both sexes | -0.1 | (-0.6,0.3) | -3.8* | (-4.0,-3.5) | -4.1* | (-4.4,-3.8) | -1.6* | (-1.9,-1.3) |
|  | male | 0.3 | (-0.1,0.7) | -2.7* | (-3.0,-2.4) | -4.2* | (-4.5,-4.0) | -1.5* | (-1.6,-1.3) |
|  | female | -0.5 | (-1.0,0.1) | -4.8* | (-5.2,-4.4) | -3.7* | (-4.0,-3.5) | -1.7* | (-2.0,-1.4) |
| **stroke** | both sexes | -2.3* | (-2.7,-2.0) | -4.1* | (-4.4,-3.7) | -2.8* | (-3.1,-2.6) | -2.0* | (-2.2,-1.9) |
|  | male | -1.5* | (-2.0,-1.0) | -2.9* | (-3.3,-2.6) | -3.0* | (-3.3,-2.7) | -1.6* | (-1.8,-1.3) |
|  | female | -2.8* | (-3.1,-2.6) | -4.8* | (-5.1,-4.5) | -2.7* | (-3.0,-2.5) | -2.3* | (-2.5,-2.2) |

*: statistically significant (p<0.05); AAPC: average annual percent change.

Table S2. The net drift value of the mortality rate of CVD, IHD and stroke attributable to secondhand smoke.

|  |  | **China** | | **Japan** | | **United States** | | **Global** | |
| --- | --- | --- | --- | --- | --- | --- | --- | --- | --- |
|  |  | Net Drift (%/year) | 95%CI | Net Drift (%/year) | 95%CI | Net Drift (%/year) | 95%CI | Net Drift (%/year) | 95%CI |
| CVD | both sexes | -2.247 | (-2.028,-2.466) | -2.632 | (-2.389,-2.875) | -3.871 | (-3.742,-4.001) | -1.969 | (-1.918,-2.019) |
|  | male | -1.030 | (-0.792,-1.267) | -1.898 | (-1.59,-2.206) | -4.026 | (-3.871,-4.18) | -1.574 | (-1.517,-1.631) |
|  | female | -3.135 | (-2.906,-3.364) | -3.698 | (-3.298,-4.097) | -3.602 | (-3.365,-3.838) | -2.340 | (-2.271,-2.408) |
| **IHD** | both sexes | -1.111 | (-0.872,-1.35) | -2.309 | (-1.996,-2.621) | -3.941 | (-3.796,-4.086) | -1.729 | (-1.682,-1.775) |
|  | male | -0.132 | (0.115,-0.379) | -1.675 | (-1.298,-2.051) | -4.120 | (-3.951,-4.289) | -1.496 | (-1.433,-1.559) |
|  | female | -1.917 | (-1.669,-2.164) | -3.433 | (-2.86,-4.002) | -3.560 | (-3.276,-3.843) | -1.984 | (-1.914,-2.055) |
| **stroke** | both sexes | -3.293 | (-3.07,-3.515) | -3.105 | (-2.715,-3.494) | -3.274 | (-2.972,-3.576) | -2.461 | (-2.387,-2.536) |
|  | male | -1.967 | (-1.719,-2.214) | -2.321 | (-1.776,-2.862) | -3.182 | (-2.775,-3.587) | -1.809 | (-1.725,-1.892) |
|  | female | -4.166 | (-3.935,-4.398) | -3.940 | (-3.375,-4.501) | -3.430 | (-2.975,-3.883) | -2.898 | (-2.819,-2.977) |

Table S3. The percent of IHD and stroke in CVD attributable to smoking in China, Japan, USA and the world from 1990 to 2017.

|  | China | | | | | Global | | | | | Japan | | | | | USA | | | | |
| --- | --- | --- | --- | --- | --- | --- | --- | --- | --- | --- | --- | --- | --- | --- | --- | --- | --- | --- | --- | --- |
| Year | CVD | IHD | stroke | IHD(%) | stroke(%) | CVD | IHD | stroke | IHD(%) | stroke(%) | CVD | IHD | stroke | IHD(%) | stroke(%) | CVD | IHD | stroke | IHD(%) | stroke(%) |
| 1990 | 46.12 | 17.31 | 28.24 | 37.53 | 61.24 | 57.71 | 37.11 | 18.94 | 64.30 | 32.82 | 37.08 | 20.25 | 14.95 | 54.61 | 40.31 | 71.07 | 57.95 | 9.82 | 81.53 | 13.81 |
| 1991 | 46.56 | 17.62 | 28.36 | 37.84 | 60.90 | 56.59 | 36.25 | 18.68 | 64.06 | 33.01 | 35.12 | 19.21 | 14.03 | 54.70 | 39.94 | 66.29 | 53.90 | 9.17 | 81.31 | 13.84 |
| 1992 | 46.74 | 17.67 | 28.48 | 37.81 | 60.92 | 55.82 | 35.62 | 18.56 | 63.80 | 33.25 | 33.40 | 18.26 | 13.27 | 54.66 | 39.73 | 61.42 | 49.81 | 8.51 | 81.10 | 13.86 |
| 1993 | 46.83 | 17.66 | 28.57 | 37.71 | 61.01 | 55.93 | 35.63 | 18.64 | 63.71 | 33.33 | 31.57 | 17.19 | 12.53 | 54.44 | 39.69 | 59.08 | 47.81 | 8.23 | 80.92 | 13.93 |
| 1994 | 46.68 | 17.52 | 28.56 | 37.53 | 61.18 | 55.27 | 35.10 | 18.53 | 63.51 | 33.53 | 29.18 | 15.73 | 11.65 | 53.91 | 39.94 | 55.69 | 44.92 | 7.82 | 80.67 | 14.03 |
| 1995 | 46.92 | 17.49 | 28.83 | 37.27 | 61.43 | 54.03 | 34.11 | 18.30 | 63.13 | 33.87 | 28.19 | 14.97 | 11.45 | 53.10 | 40.63 | 53.10 | 42.69 | 7.52 | 80.39 | 14.17 |
| 1996 | 47.14 | 17.47 | 29.06 | 37.07 | 61.63 | 52.31 | 32.79 | 17.94 | 62.67 | 34.29 | 25.90 | 13.66 | 10.53 | 52.72 | 40.66 | 50.00 | 40.04 | 7.16 | 80.09 | 14.33 |
| 1997 | 47.18 | 17.41 | 29.16 | 36.91 | 61.80 | 50.75 | 31.61 | 17.59 | 62.28 | 34.66 | 24.05 | 12.62 | 9.76 | 52.46 | 40.59 | 47.18 | 37.63 | 6.84 | 79.77 | 14.51 |
| 1998 | 47.70 | 17.56 | 29.53 | 36.80 | 61.91 | 49.34 | 30.52 | 17.30 | 61.85 | 35.06 | 23.27 | 12.17 | 9.42 | 52.30 | 40.47 | 44.82 | 35.64 | 6.56 | 79.53 | 14.63 |
| 1999 | 48.00 | 17.80 | 29.59 | 37.08 | 61.65 | 48.55 | 29.94 | 17.11 | 61.67 | 35.24 | 22.35 | 11.68 | 8.99 | 52.25 | 40.23 | 43.35 | 34.41 | 6.40 | 79.38 | 14.75 |
| 2000 | 48.74 | 18.33 | 29.81 | 37.61 | 61.16 | 47.77 | 29.36 | 16.94 | 61.47 | 35.46 | 20.76 | 10.85 | 8.26 | 52.27 | 39.78 | 41.38 | 32.73 | 6.17 | 79.11 | 14.92 |
| 2001 | 48.97 | 18.74 | 29.63 | 38.27 | 60.51 | 46.78 | 28.71 | 16.65 | 61.36 | 35.58 | 19.80 | 10.35 | 7.80 | 52.27 | 39.39 | 39.60 | 31.25 | 5.95 | 78.93 | 15.03 |
| 2002 | 49.56 | 19.44 | 29.53 | 39.22 | 59.58 | 46.11 | 28.29 | 16.42 | 61.35 | 35.61 | 18.85 | 9.86 | 7.35 | 52.29 | 39.01 | 37.76 | 29.73 | 5.71 | 78.73 | 15.11 |
| 2003 | 50.46 | 20.42 | 29.45 | 40.47 | 58.35 | 45.34 | 27.81 | 16.15 | 61.35 | 35.62 | 18.50 | 9.68 | 7.16 | 52.32 | 38.72 | 35.74 | 28.06 | 5.44 | 78.51 | 15.23 |
| 2004 | 50.12 | 20.85 | 28.69 | 41.59 | 57.24 | 43.77 | 26.84 | 15.60 | 61.31 | 35.64 | 17.72 | 9.27 | 6.79 | 52.29 | 38.34 | 33.05 | 25.86 | 5.06 | 78.24 | 15.32 |
| 2005 | 48.45 | 20.74 | 27.14 | 42.80 | 56.02 | 42.73 | 26.34 | 15.08 | 61.65 | 35.28 | 17.54 | 9.19 | 6.66 | 52.37 | 37.99 | 31.61 | 24.68 | 4.87 | 78.09 | 15.41 |
| 2006 | 45.87 | 20.24 | 25.07 | 44.13 | 54.66 | 40.76 | 25.26 | 14.23 | 61.97 | 34.91 | 16.67 | 8.74 | 6.26 | 52.39 | 37.56 | 29.65 | 23.09 | 4.61 | 77.88 | 15.56 |
| 2007 | 44.42 | 20.08 | 23.80 | 45.19 | 53.57 | 39.45 | 24.56 | 13.66 | 62.24 | 34.61 | 16.13 | 8.45 | 6.00 | 52.37 | 37.20 | 27.54 | 21.37 | 4.32 | 77.60 | 15.68 |
| 2008 | 44.00 | 20.31 | 23.14 | 46.16 | 52.59 | 38.68 | 24.15 | 13.31 | 62.44 | 34.40 | 15.57 | 8.16 | 5.72 | 52.43 | 36.76 | 25.94 | 20.10 | 4.09 | 77.47 | 15.75 |
| 2009 | 44.06 | 20.73 | 22.77 | 47.05 | 51.69 | 37.70 | 23.57 | 12.95 | 62.50 | 34.33 | 15.10 | 7.93 | 5.49 | 52.49 | 36.33 | 24.39 | 18.84 | 3.86 | 77.26 | 15.83 |
| 2010 | 44.41 | 21.23 | 22.62 | 47.81 | 50.93 | 37.10 | 23.22 | 12.70 | 62.60 | 34.23 | 14.79 | 7.78 | 5.31 | 52.59 | 35.89 | 22.75 | 17.52 | 3.61 | 77.02 | 15.88 |
| 2011 | 44.23 | 21.45 | 22.22 | 48.48 | 50.23 | 36.24 | 22.73 | 12.36 | 62.73 | 34.09 | 14.54 | 7.67 | 5.16 | 52.72 | 35.47 | 22.00 | 16.92 | 3.51 | 76.92 | 15.96 |
| 2012 | 43.67 | 21.30 | 21.80 | 48.77 | 49.92 | 35.46 | 22.25 | 12.07 | 62.75 | 34.05 | 13.95 | 7.36 | 4.90 | 52.72 | 35.10 | 21.06 | 16.16 | 3.38 | 76.76 | 16.03 |
| 2013 | 42.43 | 20.95 | 20.92 | 49.36 | 49.30 | 34.43 | 21.64 | 11.67 | 62.85 | 33.90 | 13.50 | 7.12 | 4.69 | 52.78 | 34.75 | 20.30 | 15.55 | 3.26 | 76.63 | 16.09 |
| 2014 | 42.59 | 21.06 | 20.95 | 49.46 | 49.19 | 33.83 | 21.19 | 11.53 | 62.65 | 34.08 | 13.02 | 6.86 | 4.49 | 52.70 | 34.52 | 19.65 | 15.02 | 3.18 | 76.46 | 16.17 |
| 2015 | 43.11 | 21.37 | 21.15 | 49.57 | 49.05 | 33.70 | 21.11 | 11.49 | 62.63 | 34.09 | 12.53 | 6.59 | 4.30 | 52.59 | 34.37 | 19.09 | 14.57 | 3.09 | 76.32 | 16.21 |
| 2016 | 42.72 | 21.08 | 21.04 | 49.34 | 49.24 | 33.16 | 20.71 | 11.35 | 62.44 | 34.24 | 12.48 | 6.56 | 4.31 | 52.52 | 34.50 | 18.84 | 14.36 | 3.05 | 76.23 | 16.18 |
| 2017 | 41.41 | 20.41 | 20.39 | 49.30 | 49.25 | 32.33 | 20.17 | 11.07 | 62.39 | 34.24 | 12.47 | 6.54 | 4.31 | 52.45 | 34.57 | 18.43 | 14.03 | 2.98 | 76.13 | 16.17 |

Table S4. The percent of IHD and stroke in CVD attributable to secondhand smoke in China, Japan, USA and the world from 1990 to 2017.

|  | China | | | | | Global | | | | | Japan | | | | | USA | | | | |
| --- | --- | --- | --- | --- | --- | --- | --- | --- | --- | --- | --- | --- | --- | --- | --- | --- | --- | --- | --- | --- |
| Year | CVD | IHD | stroke | IHD(%) | stroke(%) | CVD | IHD | stroke | IHD(%) | stroke(%) | CVD | IHD | stroke | IHD(%) | stroke(%) | CVD | IHD | stroke | IHD(%) | stroke(%) |
| 1990 | 14.55 | 6.03 | 8.52 | 41.43 | 58.57 | 11.81 | 7.55 | 4.26 | 63.96 | 36.04 | 4.70 | 2.84 | 1.86 | 60.40 | 39.60 | 6.08 | 5.36 | 0.72 | 88.10 | 11.90 |
| 1991 | 14.35 | 5.99 | 8.36 | 41.72 | 58.28 | 11.62 | 7.43 | 4.19 | 63.94 | 36.06 | 4.49 | 2.72 | 1.77 | 60.50 | 39.50 | 5.83 | 5.13 | 0.70 | 87.94 | 12.06 |
| 1992 | 14.02 | 5.86 | 8.16 | 41.79 | 58.21 | 11.50 | 7.35 | 4.15 | 63.92 | 36.08 | 4.28 | 2.59 | 1.69 | 60.53 | 39.47 | 5.54 | 4.86 | 0.68 | 87.78 | 12.22 |
| 1993 | 13.80 | 5.76 | 8.04 | 41.74 | 58.26 | 11.49 | 7.35 | 4.14 | 63.94 | 36.06 | 4.07 | 2.45 | 1.61 | 60.38 | 39.62 | 5.45 | 4.77 | 0.68 | 87.59 | 12.41 |
| 1994 | 13.55 | 5.63 | 7.91 | 41.57 | 58.43 | 11.38 | 7.27 | 4.11 | 63.85 | 36.15 | 3.77 | 2.26 | 1.51 | 59.95 | 40.05 | 5.23 | 4.57 | 0.66 | 87.36 | 12.64 |
| 1995 | 13.21 | 5.47 | 7.74 | 41.44 | 58.56 | 11.14 | 7.10 | 4.04 | 63.75 | 36.25 | 3.66 | 2.17 | 1.49 | 59.21 | 40.79 | 5.05 | 4.40 | 0.65 | 87.14 | 12.86 |
| 1996 | 12.97 | 5.36 | 7.61 | 41.35 | 58.65 | 10.86 | 6.90 | 3.96 | 63.57 | 36.43 | 3.37 | 1.99 | 1.38 | 59.01 | 40.99 | 4.79 | 4.16 | 0.63 | 86.88 | 13.12 |
| 1997 | 12.68 | 5.24 | 7.43 | 41.36 | 58.64 | 10.61 | 6.74 | 3.88 | 63.47 | 36.53 | 3.15 | 1.85 | 1.29 | 58.95 | 41.05 | 4.53 | 3.92 | 0.61 | 86.60 | 13.40 |
| 1998 | 12.45 | 5.16 | 7.29 | 41.47 | 58.53 | 10.34 | 6.55 | 3.79 | 63.33 | 36.67 | 3.04 | 1.80 | 1.25 | 59.00 | 41.00 | 4.29 | 3.71 | 0.58 | 86.39 | 13.61 |
| 1999 | 12.20 | 5.12 | 7.08 | 41.99 | 58.01 | 10.17 | 6.45 | 3.73 | 63.37 | 36.63 | 2.93 | 1.73 | 1.20 | 59.18 | 40.82 | 4.14 | 3.57 | 0.57 | 86.22 | 13.78 |
| 2000 | 12.23 | 5.23 | 7.00 | 42.77 | 57.23 | 10.02 | 6.35 | 3.67 | 63.35 | 36.65 | 2.73 | 1.62 | 1.11 | 59.43 | 40.57 | 3.93 | 3.38 | 0.55 | 86.00 | 14.00 |
| 2001 | 12.18 | 5.33 | 6.85 | 43.74 | 56.26 | 9.84 | 6.24 | 3.60 | 63.44 | 36.56 | 2.59 | 1.55 | 1.05 | 59.71 | 40.29 | 3.73 | 3.20 | 0.53 | 85.84 | 14.16 |
| 2002 | 12.30 | 5.53 | 6.78 | 44.93 | 55.07 | 9.73 | 6.19 | 3.54 | 63.57 | 36.43 | 2.47 | 1.48 | 0.99 | 59.99 | 40.01 | 3.50 | 3.00 | 0.50 | 85.71 | 14.29 |
| 2003 | 12.49 | 5.80 | 6.69 | 46.44 | 53.56 | 9.59 | 6.12 | 3.48 | 63.77 | 36.23 | 2.42 | 1.46 | 0.96 | 60.27 | 39.73 | 3.25 | 2.78 | 0.47 | 85.56 | 14.44 |
| 2004 | 12.47 | 5.96 | 6.51 | 47.79 | 52.21 | 9.30 | 5.95 | 3.36 | 63.92 | 36.08 | 2.34 | 1.42 | 0.93 | 60.47 | 39.53 | 2.96 | 2.53 | 0.43 | 85.43 | 14.57 |
| 2005 | 12.15 | 5.97 | 6.18 | 49.11 | 50.89 | 9.10 | 5.86 | 3.24 | 64.37 | 35.63 | 2.32 | 1.41 | 0.91 | 60.79 | 39.21 | 2.83 | 2.41 | 0.41 | 85.33 | 14.67 |
| 2006 | 11.51 | 5.81 | 5.70 | 50.50 | 49.50 | 8.72 | 5.66 | 3.06 | 64.89 | 35.11 | 2.23 | 1.36 | 0.87 | 61.08 | 38.92 | 2.67 | 2.27 | 0.40 | 85.17 | 14.83 |
| 2007 | 11.19 | 5.78 | 5.41 | 51.62 | 48.38 | 8.46 | 5.52 | 2.94 | 65.27 | 34.73 | 2.18 | 1.34 | 0.84 | 61.32 | 38.68 | 2.50 | 2.12 | 0.37 | 85.02 | 14.98 |
| 2008 | 11.18 | 5.88 | 5.30 | 52.59 | 47.41 | 8.32 | 5.45 | 2.87 | 65.53 | 34.47 | 2.13 | 1.31 | 0.82 | 61.65 | 38.35 | 2.39 | 2.03 | 0.36 | 84.93 | 15.07 |
| 2009 | 11.32 | 6.06 | 5.26 | 53.51 | 46.49 | 8.17 | 5.37 | 2.80 | 65.69 | 34.31 | 2.07 | 1.29 | 0.79 | 62.00 | 38.00 | 2.28 | 1.94 | 0.35 | 84.80 | 15.20 |
| 2010 | 11.44 | 6.22 | 5.22 | 54.35 | 45.65 | 8.09 | 5.33 | 2.76 | 65.91 | 34.09 | 2.04 | 1.27 | 0.77 | 62.29 | 37.71 | 2.17 | 1.84 | 0.33 | 84.72 | 15.28 |
| 2011 | 11.44 | 6.30 | 5.14 | 55.05 | 44.95 | 7.95 | 5.26 | 2.69 | 66.14 | 33.86 | 2.02 | 1.26 | 0.76 | 62.52 | 37.48 | 2.14 | 1.81 | 0.33 | 84.63 | 15.37 |
| 2012 | 11.30 | 6.26 | 5.03 | 55.45 | 44.55 | 7.82 | 5.18 | 2.64 | 66.29 | 33.71 | 1.93 | 1.21 | 0.72 | 62.76 | 37.24 | 2.10 | 1.77 | 0.32 | 84.52 | 15.48 |
| 2013 | 10.96 | 6.14 | 4.82 | 56.02 | 43.98 | 7.64 | 5.08 | 2.56 | 66.50 | 33.50 | 1.86 | 1.17 | 0.69 | 62.95 | 37.05 | 2.07 | 1.75 | 0.32 | 84.43 | 15.57 |
| 2014 | 10.92 | 6.14 | 4.78 | 56.21 | 43.79 | 7.56 | 5.03 | 2.54 | 66.46 | 33.54 | 1.79 | 1.12 | 0.66 | 62.99 | 37.01 | 2.05 | 1.73 | 0.32 | 84.29 | 15.71 |
| 2015 | 10.90 | 6.14 | 4.76 | 56.32 | 43.68 | 7.55 | 5.02 | 2.53 | 66.54 | 33.46 | 1.70 | 1.07 | 0.63 | 62.99 | 37.01 | 2.06 | 1.73 | 0.32 | 84.20 | 15.80 |
| 2016 | 10.68 | 6.00 | 4.68 | 56.21 | 43.79 | 7.46 | 4.96 | 2.50 | 66.51 | 33.49 | 1.67 | 1.05 | 0.62 | 62.85 | 37.15 | 2.09 | 1.76 | 0.33 | 84.16 | 15.84 |
| 2017 | 10.26 | 5.77 | 4.49 | 56.22 | 43.78 | 7.29 | 4.86 | 2.44 | 66.58 | 33.42 | 1.65 | 1.04 | 0.62 | 62.73 | 37.27 | 2.11 | 1.78 | 0.34 | 84.12 | 15.88 |
