# Supplementary material for: A cross country comparison for the burden of cardiovascular disease attributable to tobacco exposure in China, Japan, USA and world
Source: BMC Public Health. 2020 Jun 8;20:888. doi: 10.1186/s12889-020-09031-7 (PMC7282071; doi:10.1186/s12889-020-09031-7)
Supplement: Supplementary file 2 — Additional file 2: Figure S1. The ASMR of IHD attributable to smoking in China, Japan, USA and the world from 1990 to 2017. Figure S2. The ASMR of stroke attributable to smoking in China, Japan, USA and the world from 1990 to 2017. Figure S3. The ASMR of CVD attributable to secondhand smoke in China, Japan, USA and the world from 1990 to 2017. Figure S4. The ASMR of IHD attributable to secondhand smoke in China, Japan, USA and the world from 1990 to 2017. Figure S5. The ASMR of stroke attributable to secondhand smoke in China, Japan, USA and the world from 1990 to 2017. Figure S6. The APC results of IHD attributable to smoking in China, Japan, USA and the world from 1990 to 2017. Figure S7. The APC results of stroke attributable to smoking in China, Japan, USA and the world from 1990 to 2017. Figure S8. The local drift with net drift values of the mortality rate of CVD, IHD and stroke attributable to smoking. Figure S9. The APC results of CVD attributable to secondhand smoke in China, Japan, USA and the world from 1990 to 2017. Figure S10. The APC results of IHD attributable to secondhand smoke in China, Japan, USA and the world from 1990 to 2017. Figure S11. The APC results of stroke attributable to secondhand smoke in China, Japan, USA and the world from 1990 to 2017. Figure S12. The local drift with net drift values of the mortality rate of CVD, IHD and stroke attributable to secondhand smoke. [file 12889_2020_9031_MOESM2_ESM.pdf]

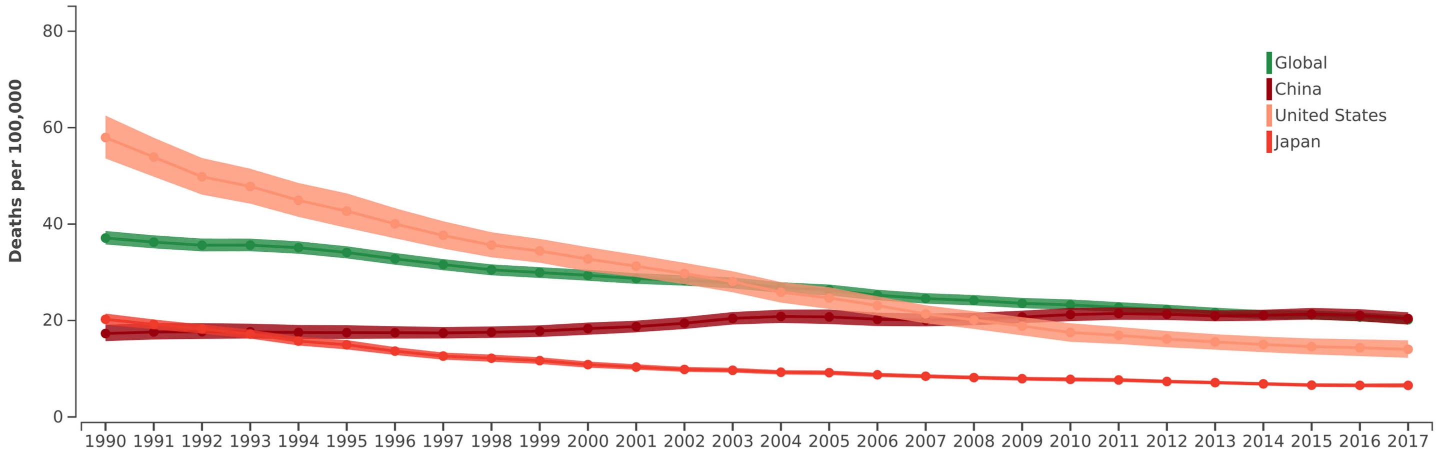

Figure S1(A). The mortality rate of IHD attributable to smoking for both sexs in China, Japan, USA and the world from 1990 to 2017.

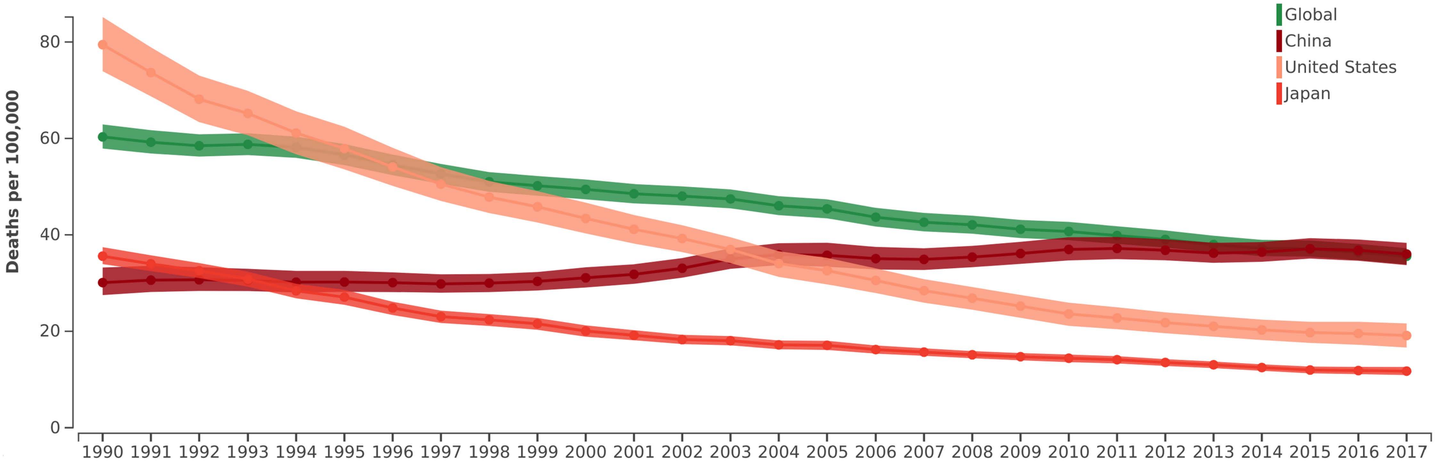

Figure S1(B). The mortality rate of IHD attributable to smoking for male in China, Japan, USA and the world from 1990 to 2017.

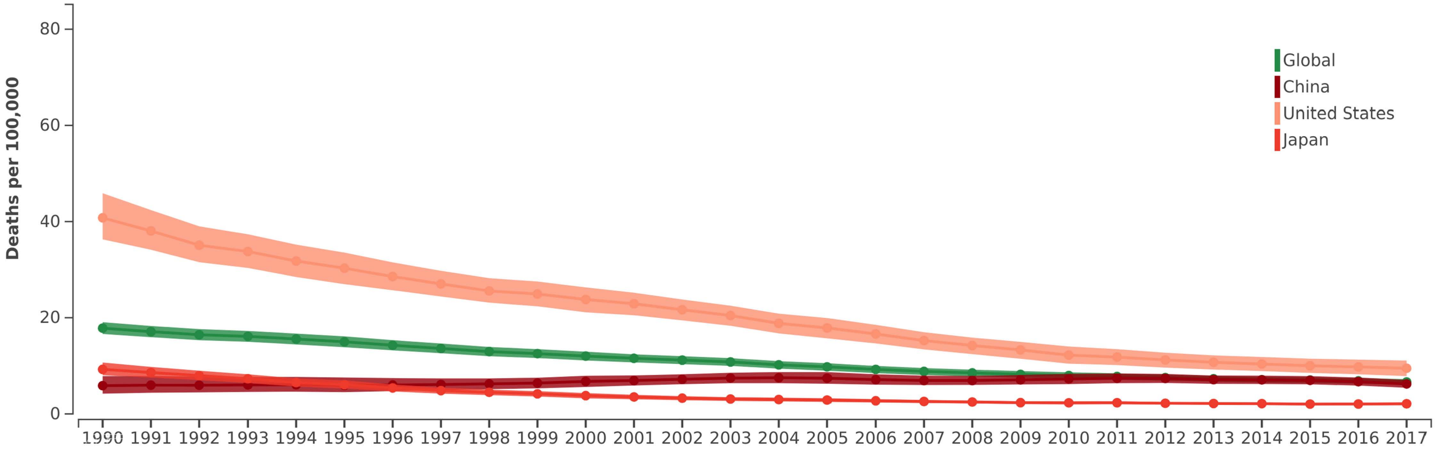

Figure S1(C). The mortality rate of IHD attributable to smoking for female in China, Japan, USA and the world from 1990 to 2017.

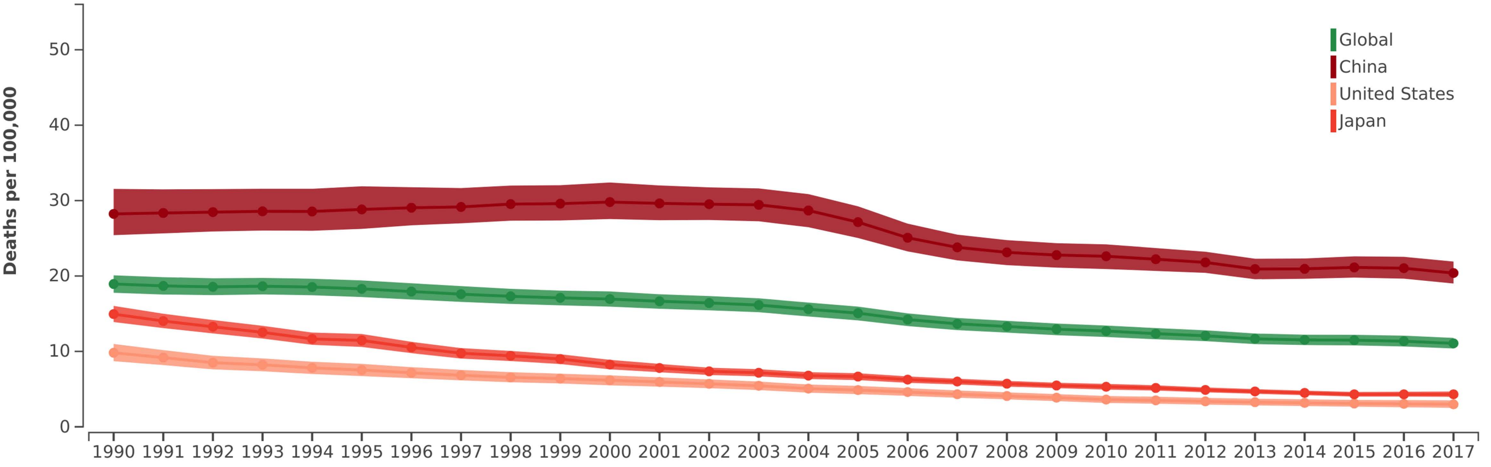

Figure S2(A). The mortality rate of stroke attributable to smoking for both sexes in China, Japan, USA, and the world from 1990 to 2017

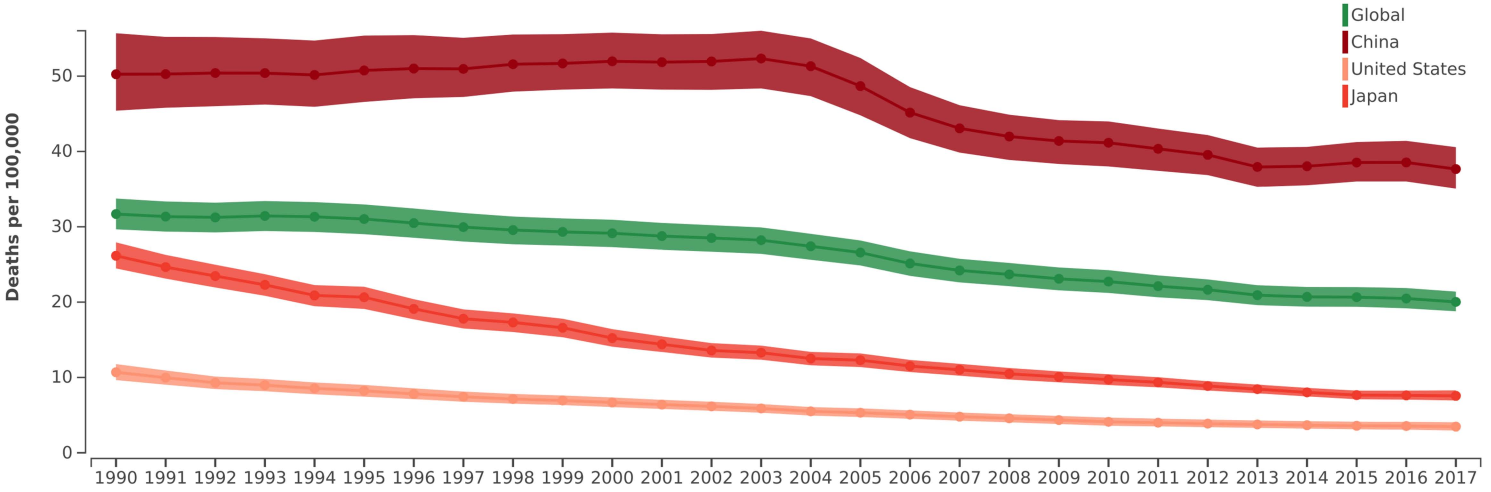

Figure S2(B). The mortality rate of stroke attributable to smoking for male in China, Japan, USA, and the world from 1990 to 2017

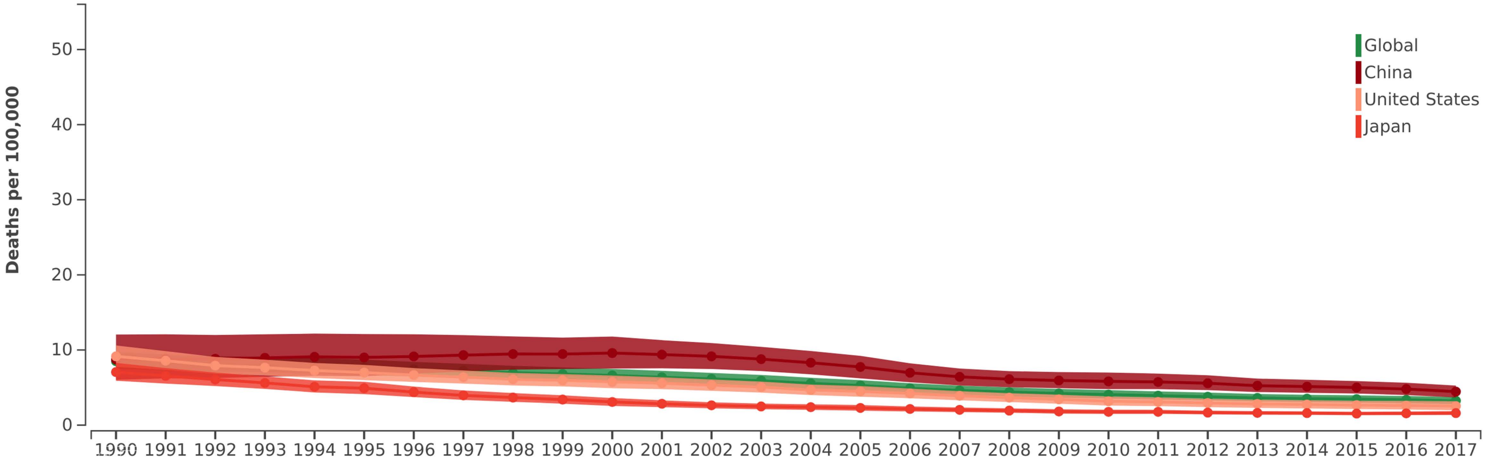

Figure S2(C). The mortality rate of stroke attributable to smoking for male in China, Japan, USA, and the world from 1990 to 2017

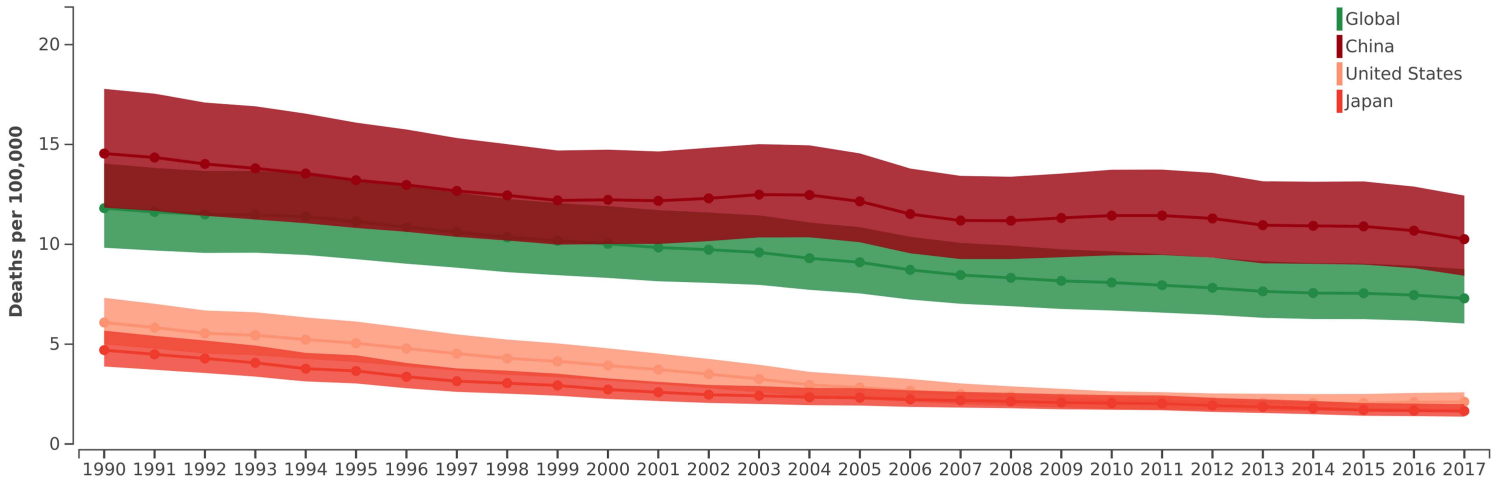

Figure S3A. The ASMR of CVD attributable to secondhand smoke for both sexes in China, Japan, USA and the world from 1990 to 2017.

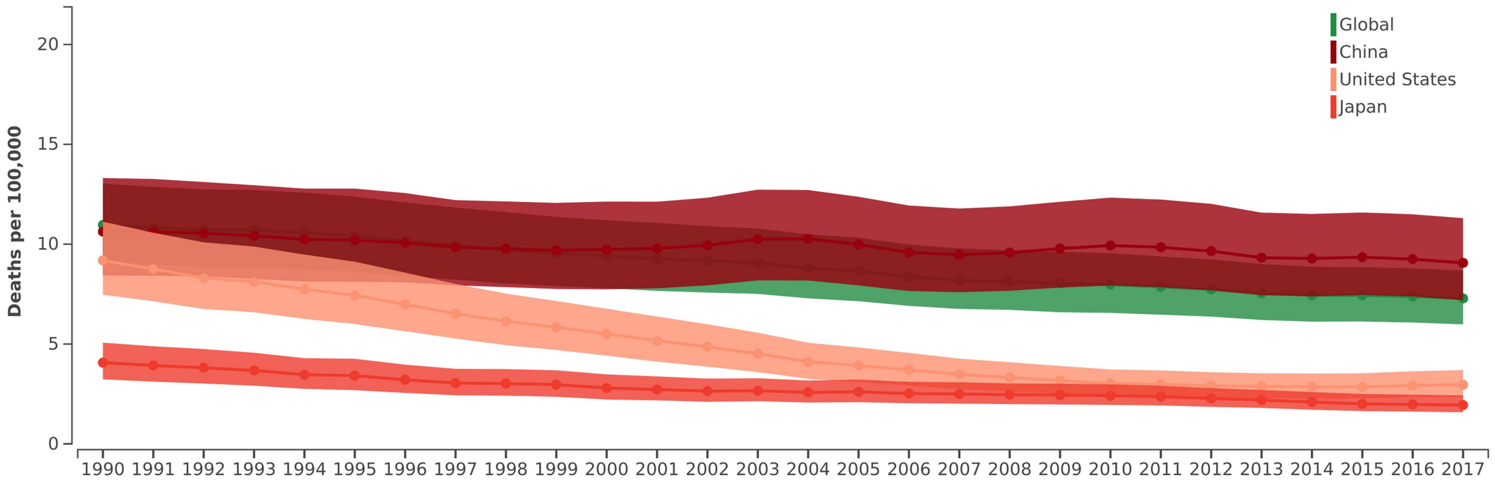

Figure S3B. The ASMR of CVD attributable to secondhand smoke for male in China, Japan, USA and the world from 1990 to 2017.

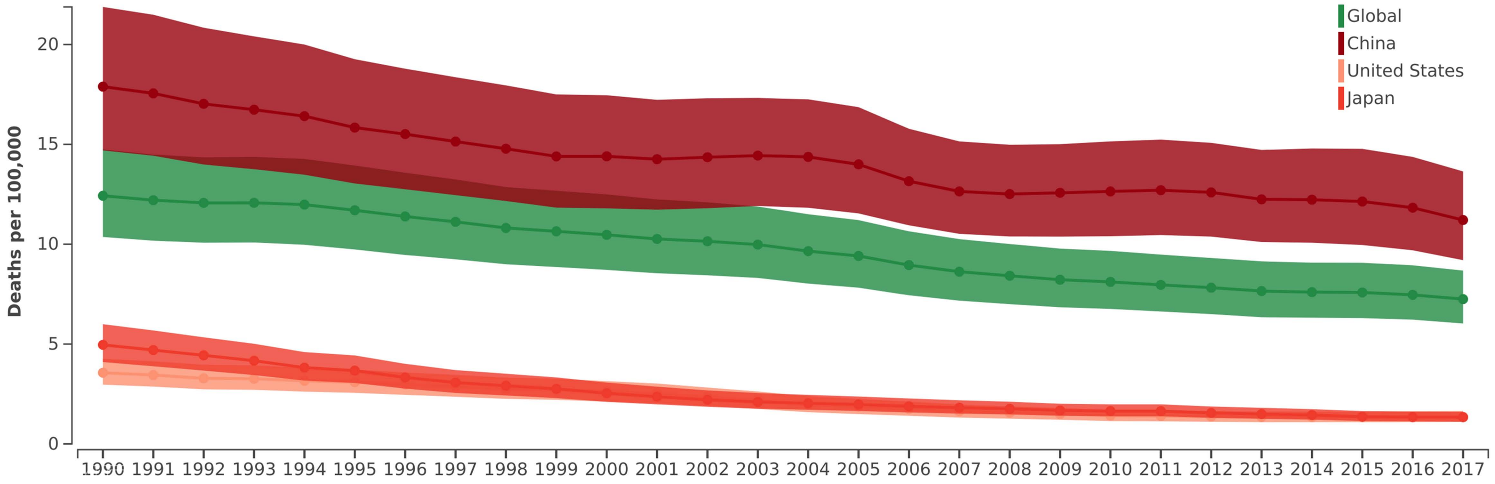

Figure S3C. The ASMR of CVD attributable to secondhand smoke for female in China, Japan, USA and the world from 1990 to 2017.

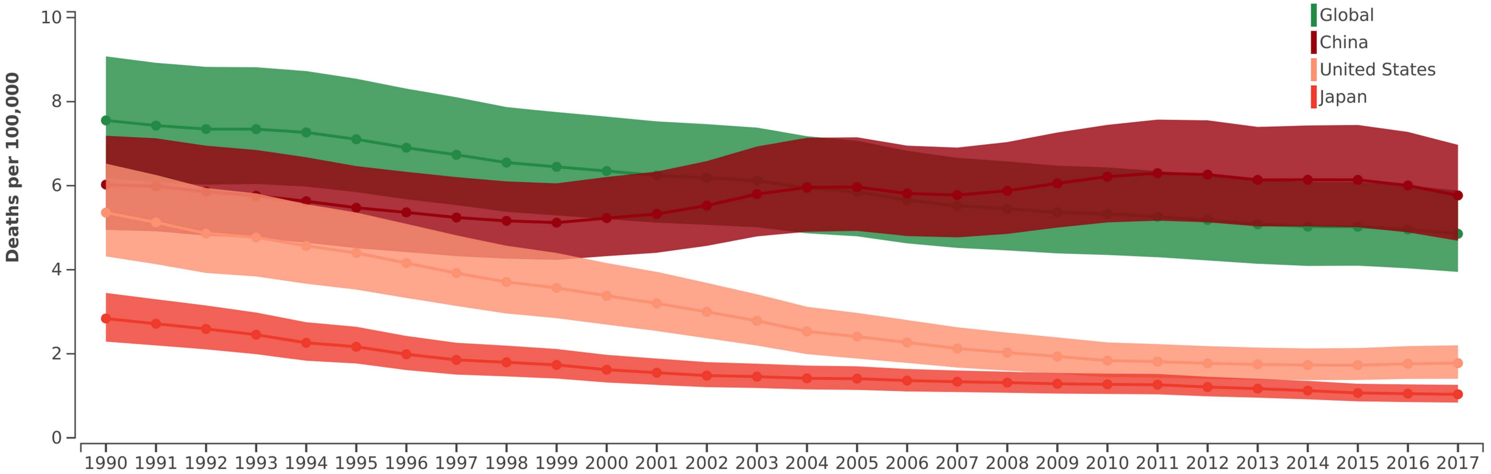

Figure S4(A). The mortality rate of IHD attributable to secondhand smoke for both sexs in China, Japan, USA and the world from 1990 to 2017.

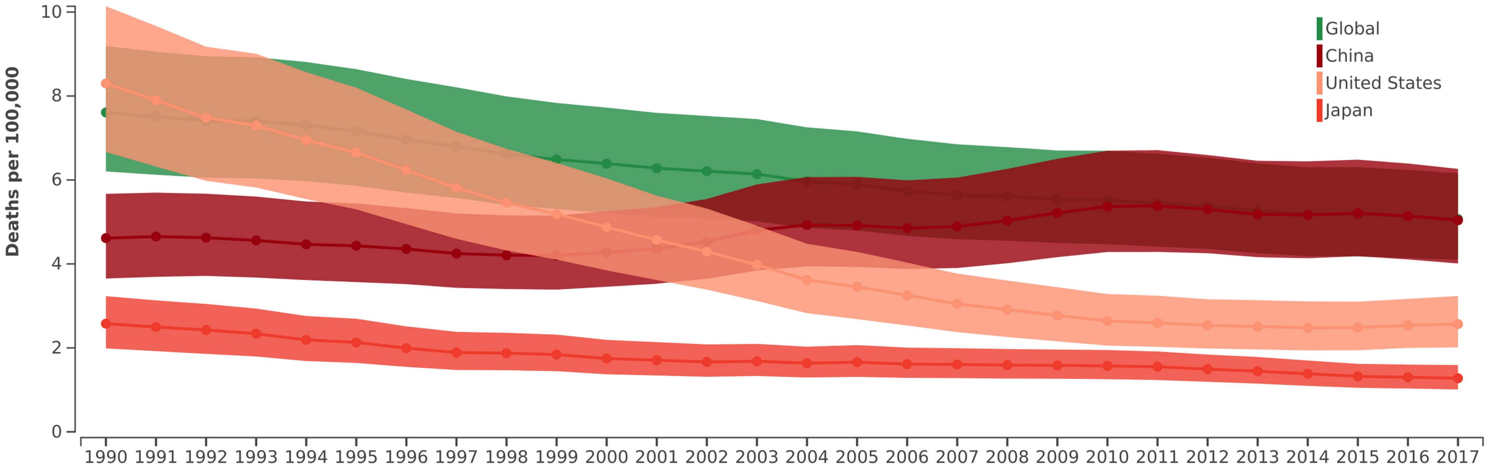

Figure S4(B). The mortality rate of IHD attributable to secondhand smoke for male in China, Japan, USA and the world from 1990 to 2017.

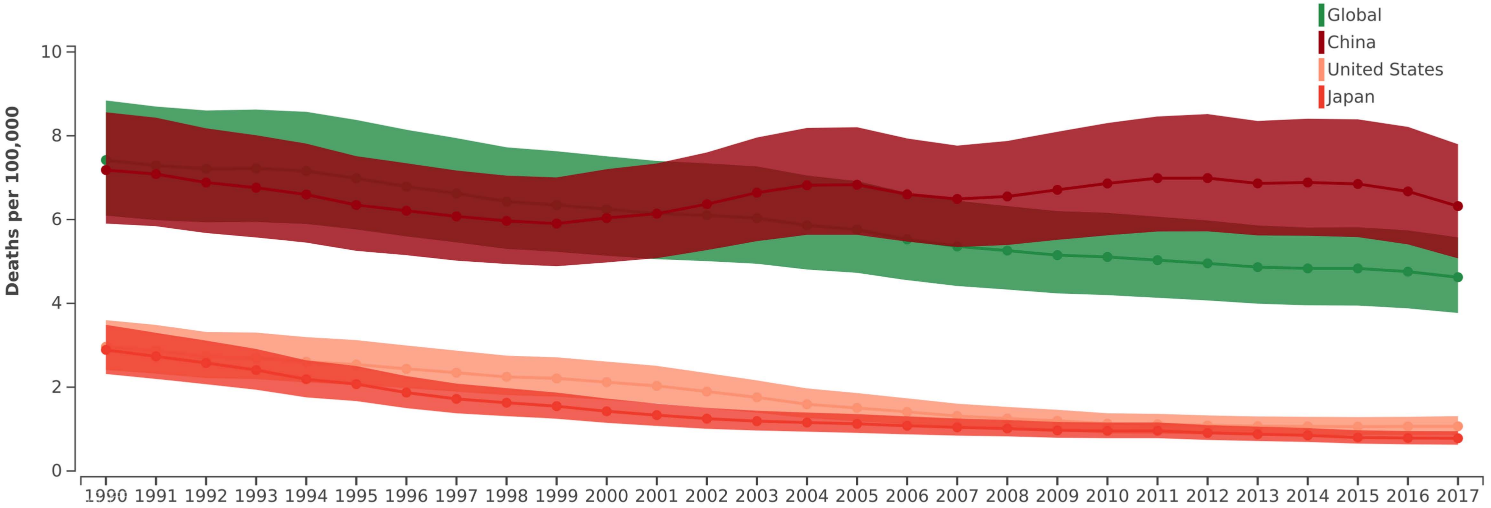

Figure S4(C). The mortality rate of IHD attributable to secondhand smoke for female in China, Japan, USA and the world from 1990 to 2017.

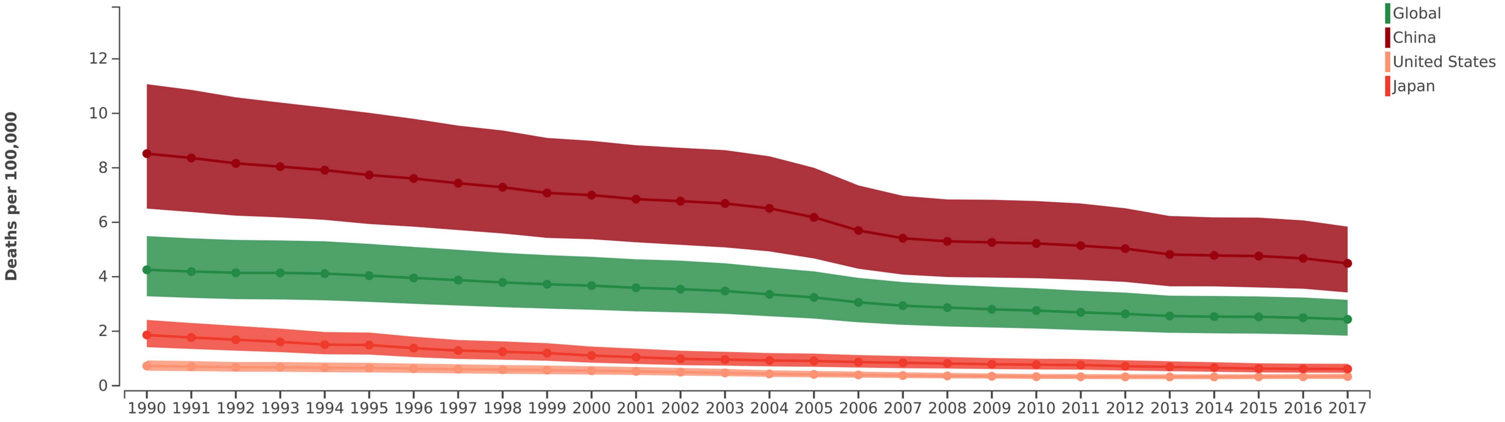

Figure S5(A). The mortality rate of stroke attributable to secondhand smoke for both sexs in China, Japan, USA and the world from 1990 to 2017.

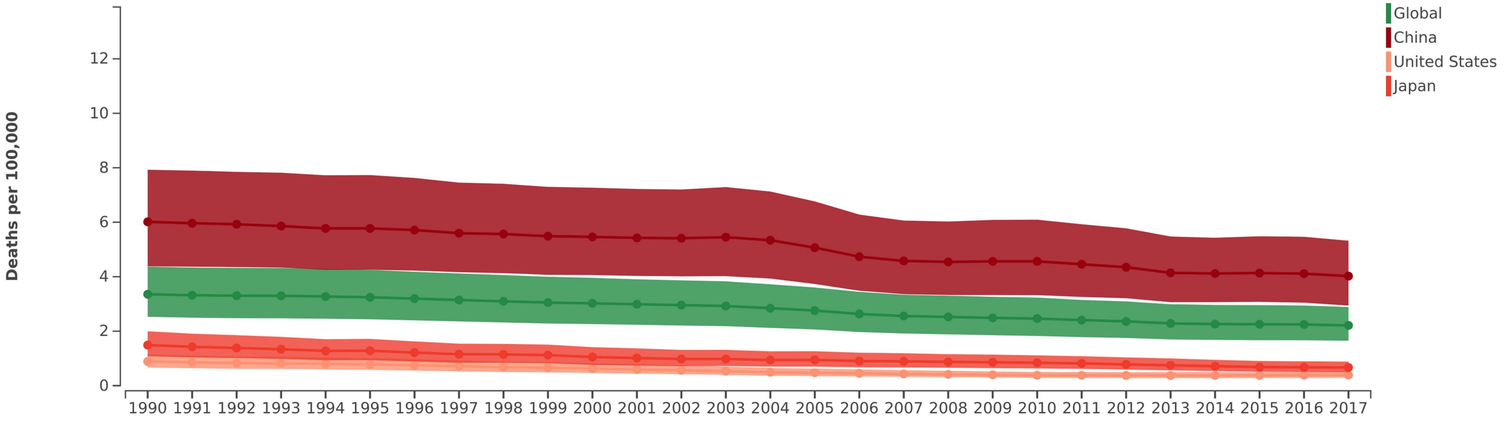

Figure S5(B). The mortality rate of stroke attributable to secondhand smoke for male in China, Japan, USA and the world from 1990 to 2017.

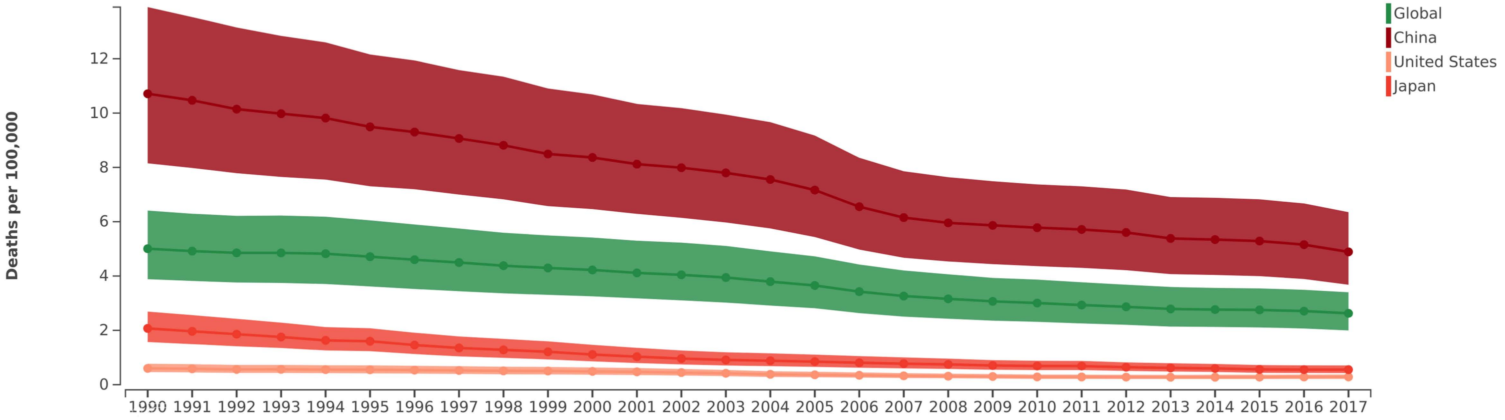

Figure S5(C). The mortality rate of stroke attributable to secondhand smoke for female in China, Japan, USA and the world from 1990 to 2017.

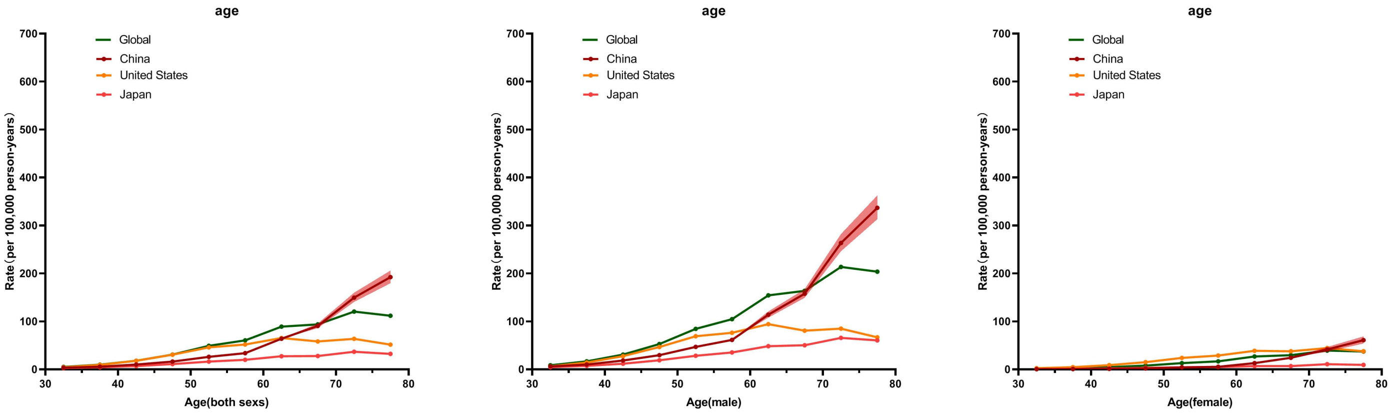

Figure S6(A). the longitudinal age curves of the mortality rate of IHD attributable to smoking in global, China, United States and Japan.

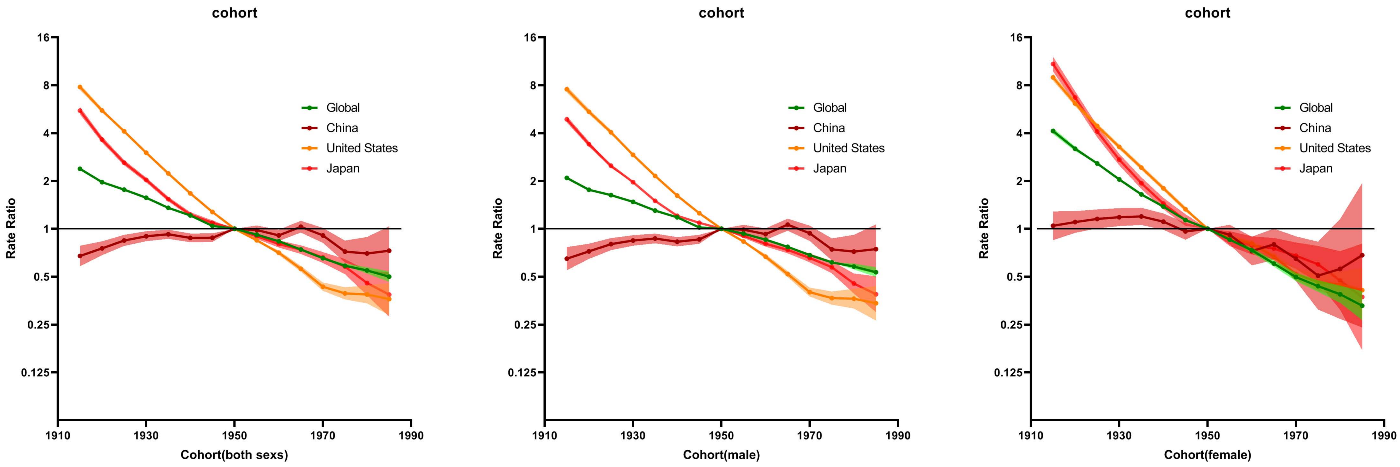

Figure S6(B). The estimated cohort RRs of the mortality rate of IHD attributable to smoking in global, China, United States and Japan.

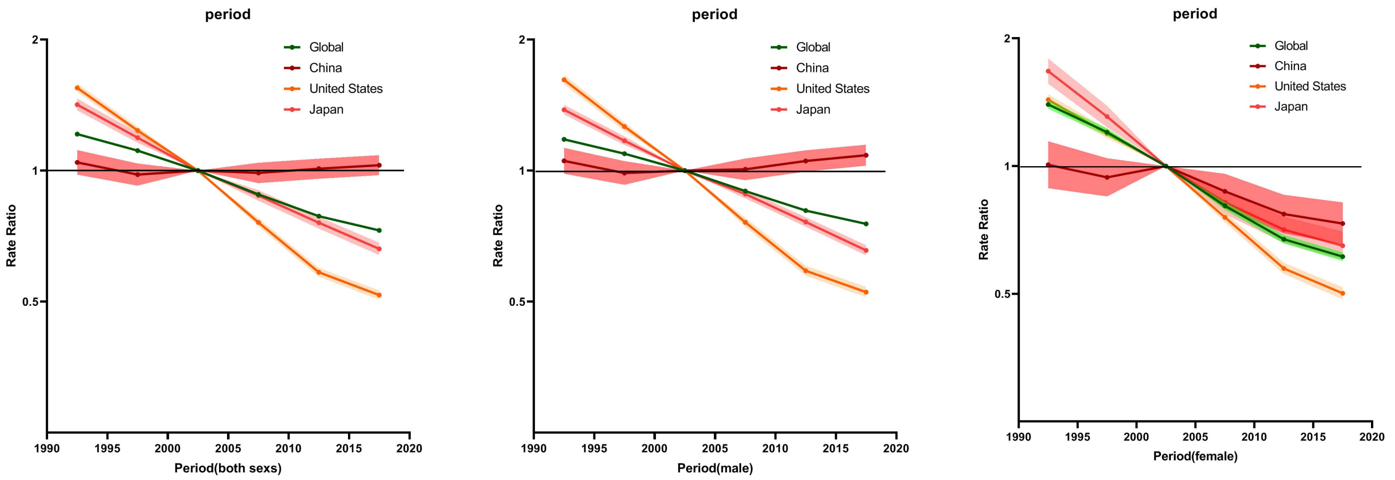

Figure S6(C). The estimated period RRs of the mortality rate of IHD attributable to smoking in global, China, United States and Japan.

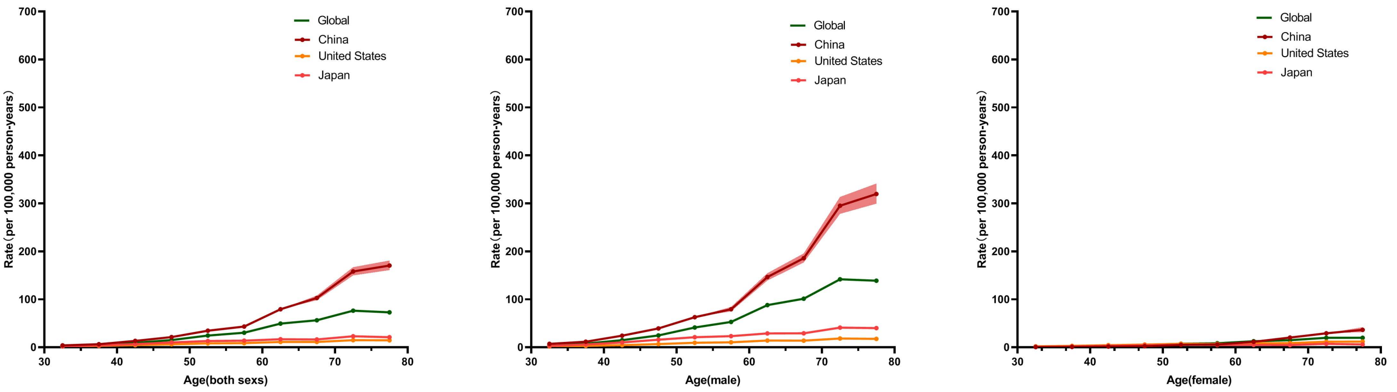

Figure S7(A). the longitudinal age curves of the mortality rate of stroke attributable to smoking in global, China, United States and Japan.

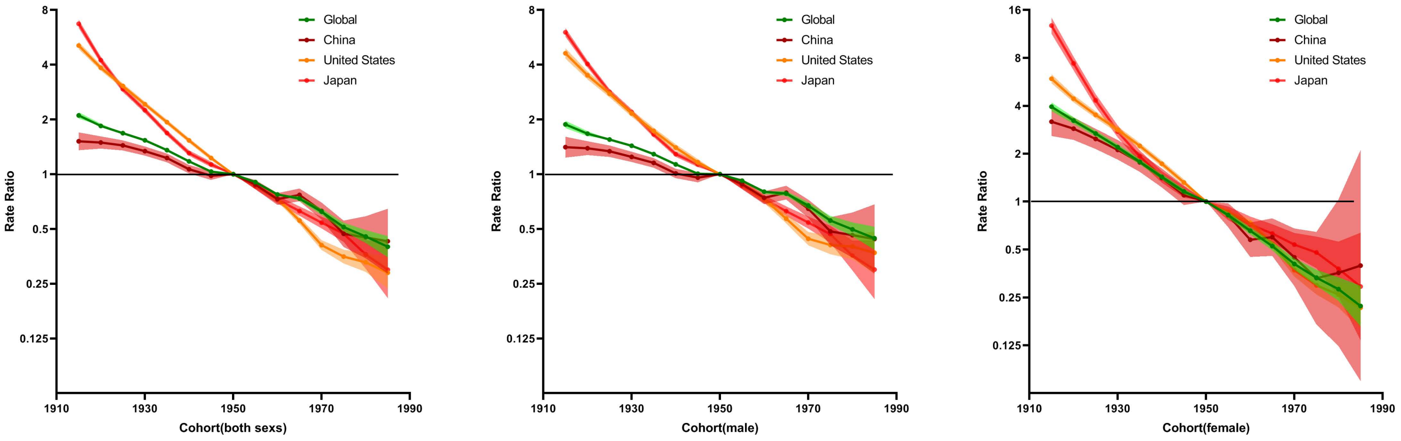

Figure S7(B). The estimated cohort RRs of the mortality rate of stroke attributable to smoking in global, China, United States and Japan.

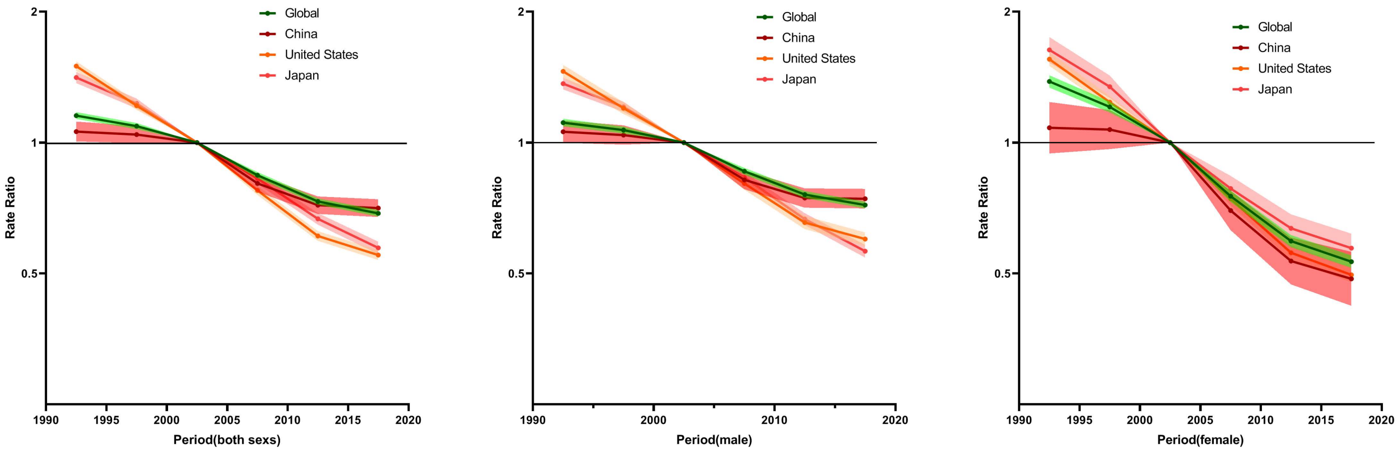

Figure S7(C). The estimated period RRs of the mortality rate of stroke attributable to smoking in global, China, United States and Japan.

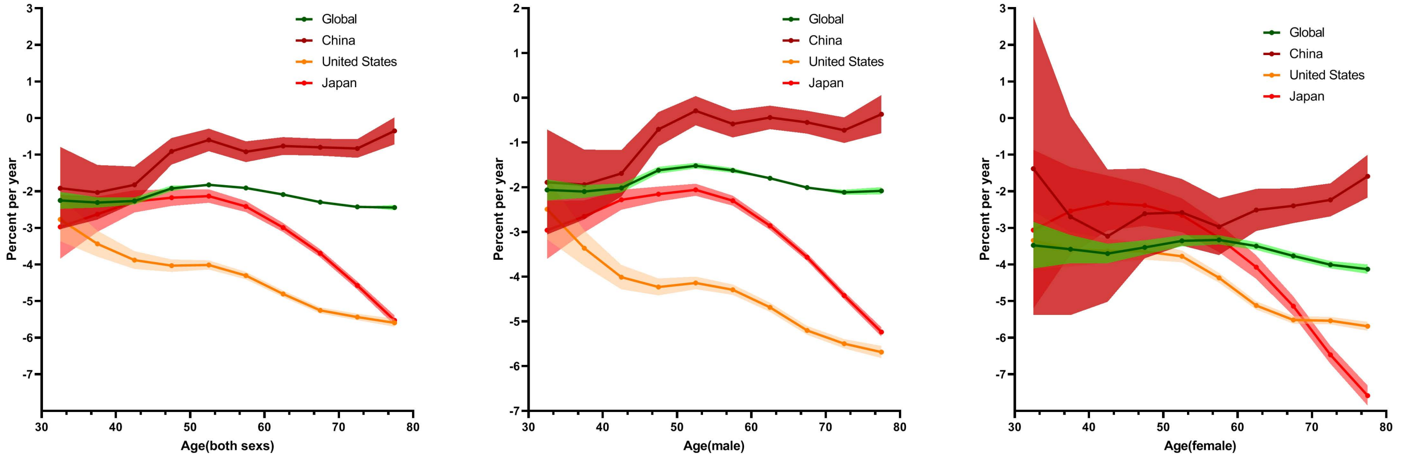

Figure S8(A). The local drift with net drift values of the mortality rate of CVD attributable to smoking.

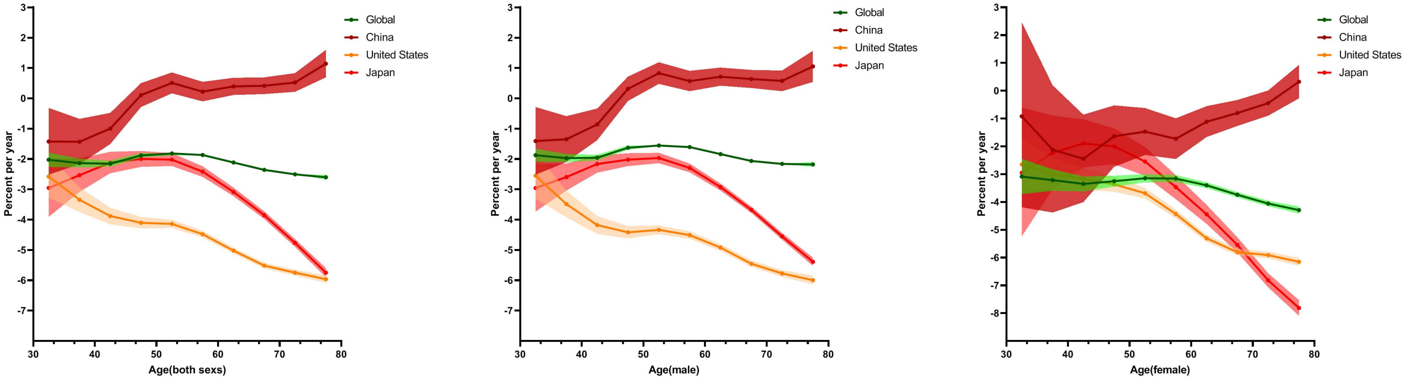

Figure S8(B). The local drift with net drift values of the mortality rate of IHD attributable to smoking.

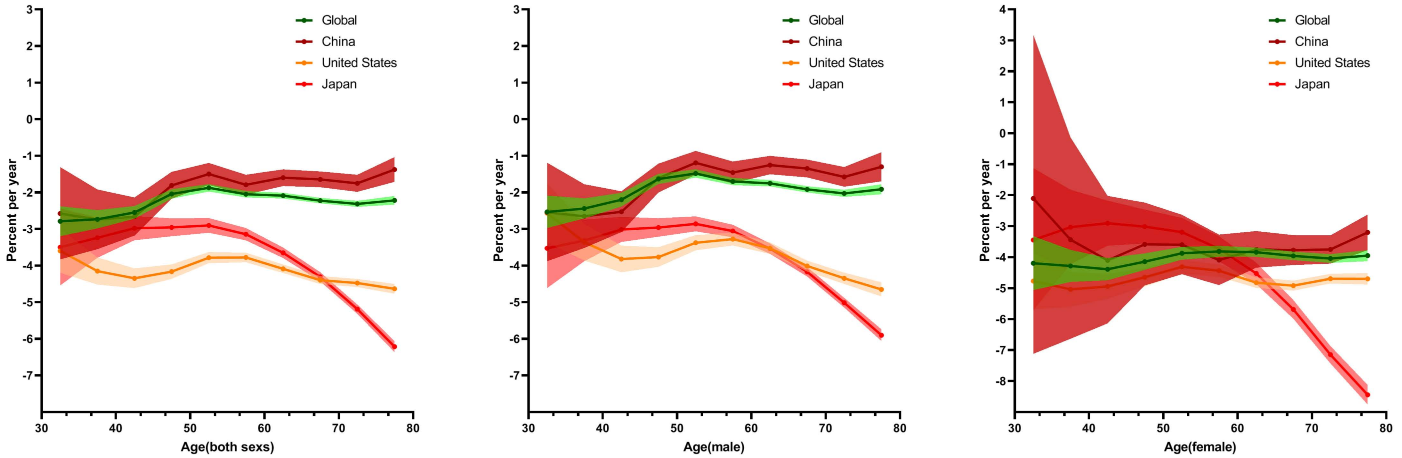

Figure S8(C). The local drift with net drift values of the mortality rate of stroke attributable to smoking.

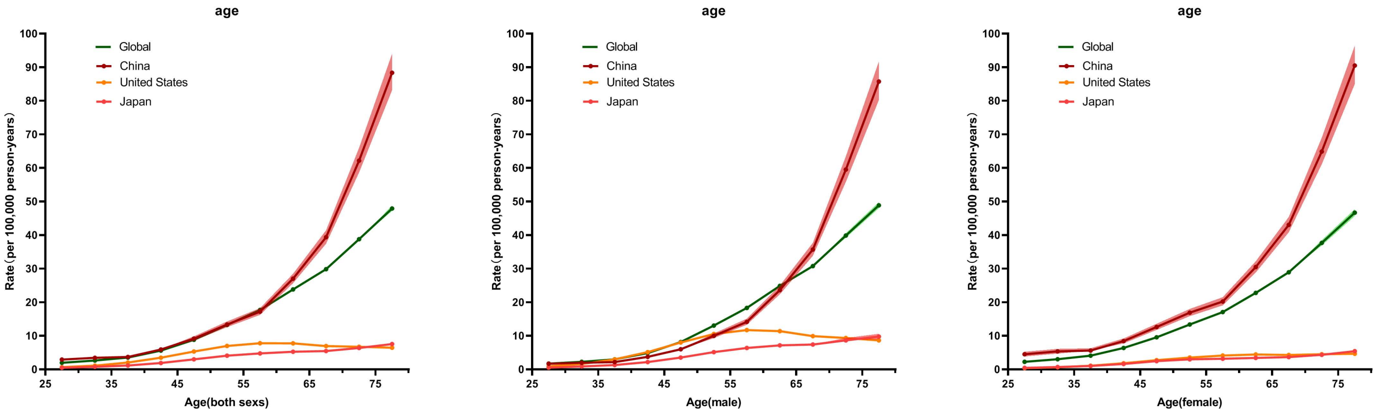

Figure S9(A). the longitudinal age curves of the mortality rate of CVD attributable to secondhand smoke in global, China, United States and Japan.

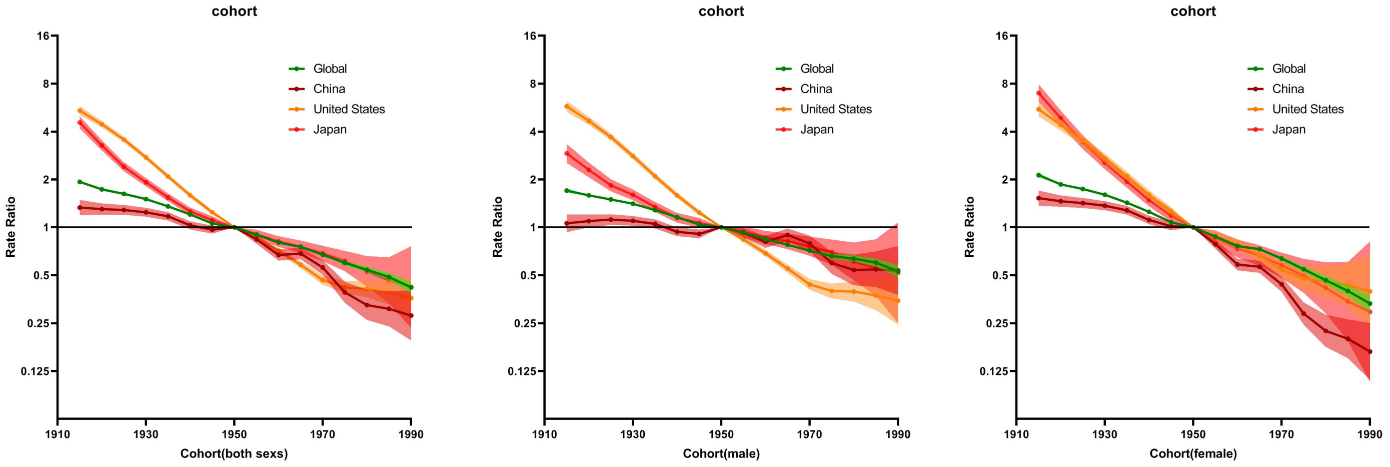

Figure S9(B). The estimated cohort RRs of of the mortality rate of CVD attributable to secondhand smoke in global, China, United States and Japan.

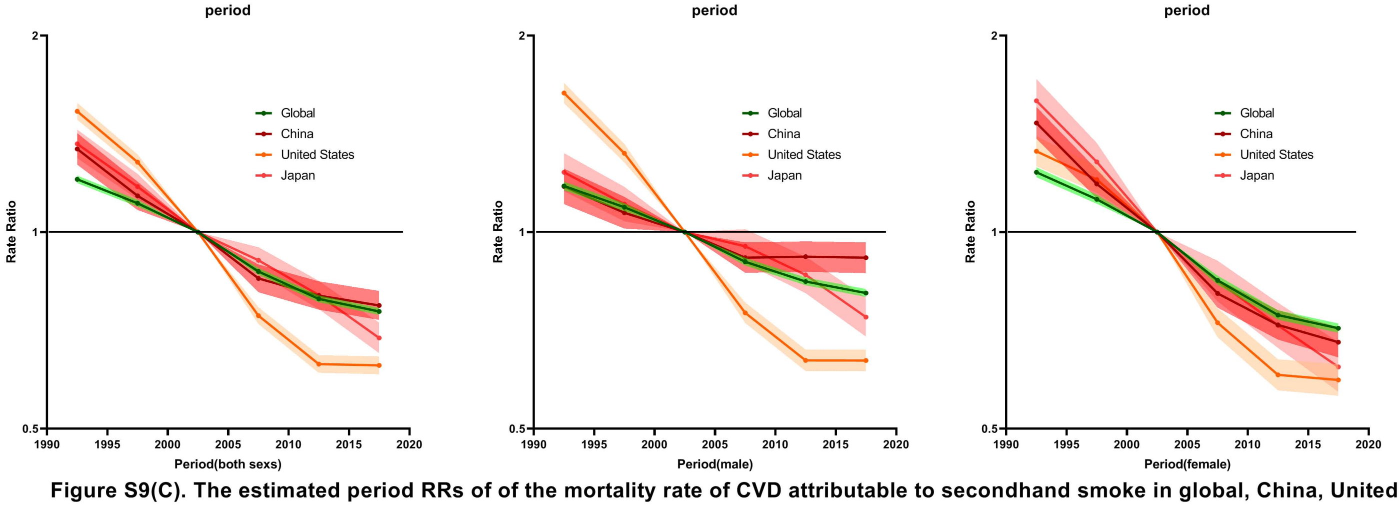

Figure S9(C). The estimated period RRs of of the mortality rate of CVD attributable to secondhand smoke in global, China, United States and Japan.

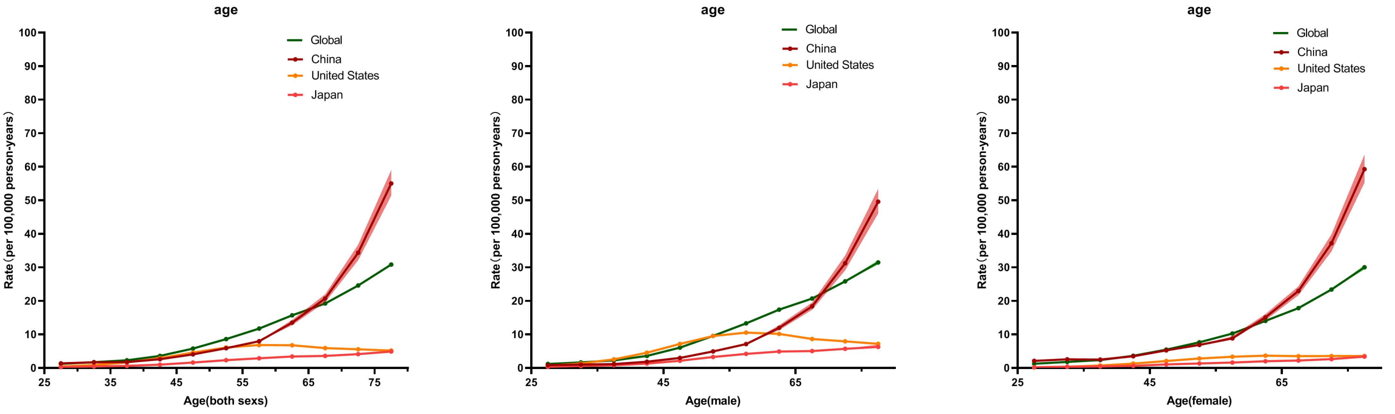

Figure S10(A). the longitudinal age curves of the mortality rate of IHD attributable to secondhand smoke in global, China, United States and Japan.

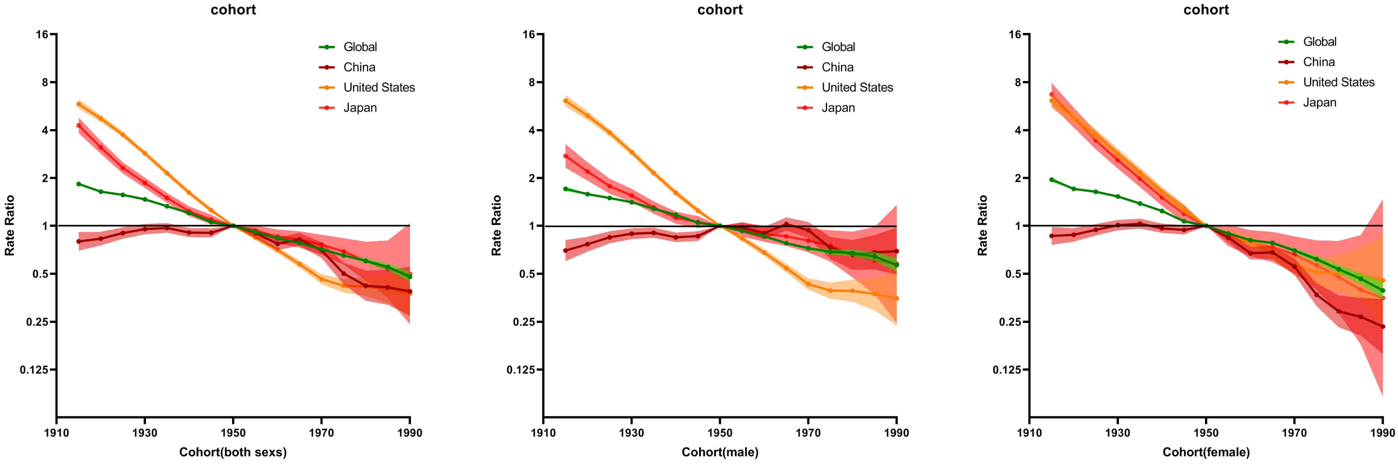

Figure S10(B). The estimated cohort RRs of the mortality rate of IHD attributable to secondhand smoke in global, China, United States and Japan.

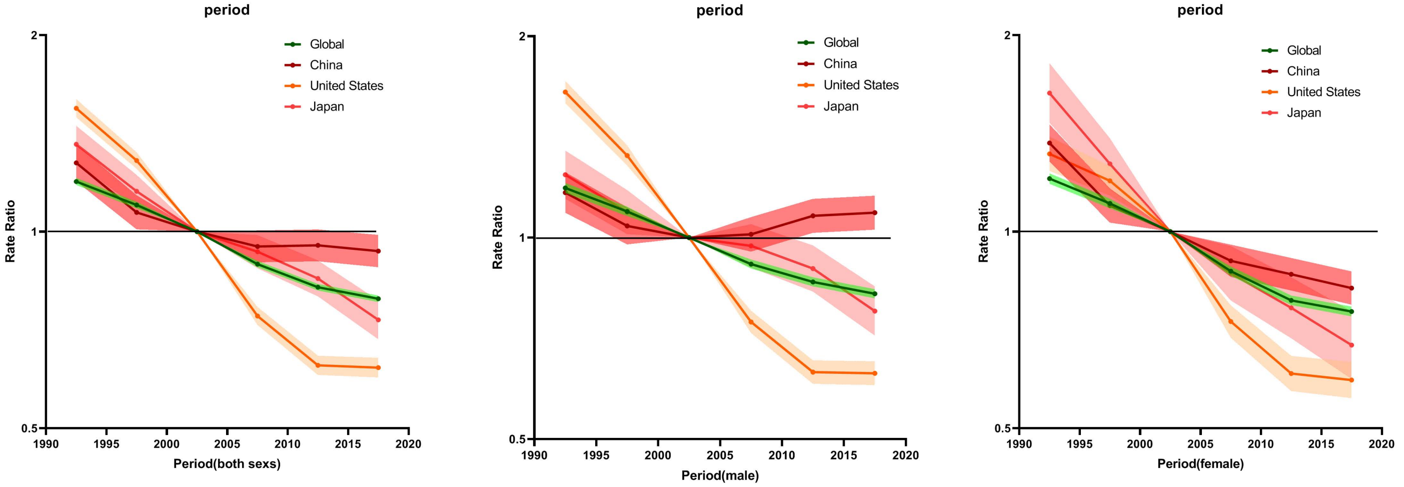

Figure S10(C). The estimated period RRs of the mortality rate of IHD attributable to secondhand smoke in global, China, United States and Japan.

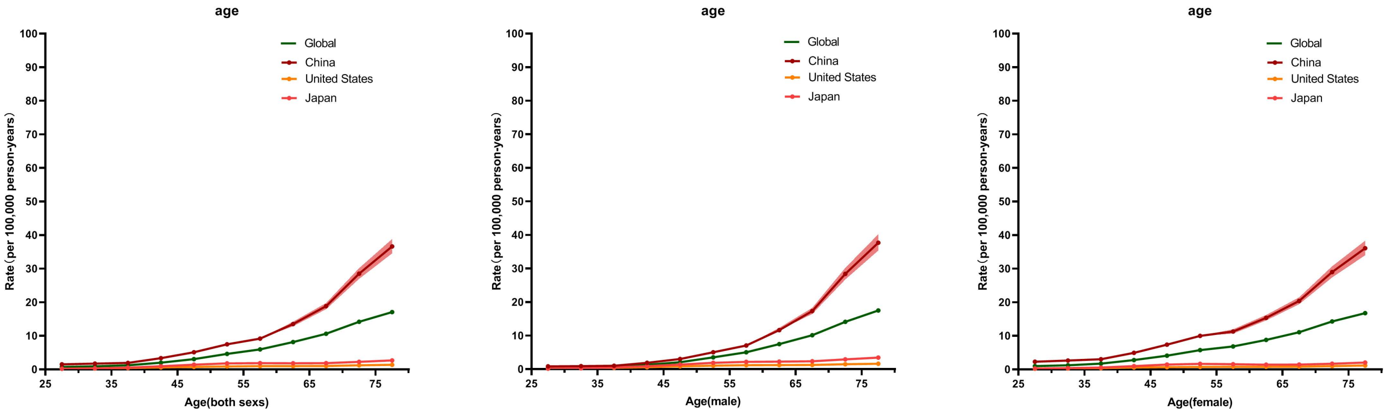

Figure S11(A). the longitudinal age curves of the mortality rate of stroke attributable to secondhand smoke in global, China, United States and Japan.

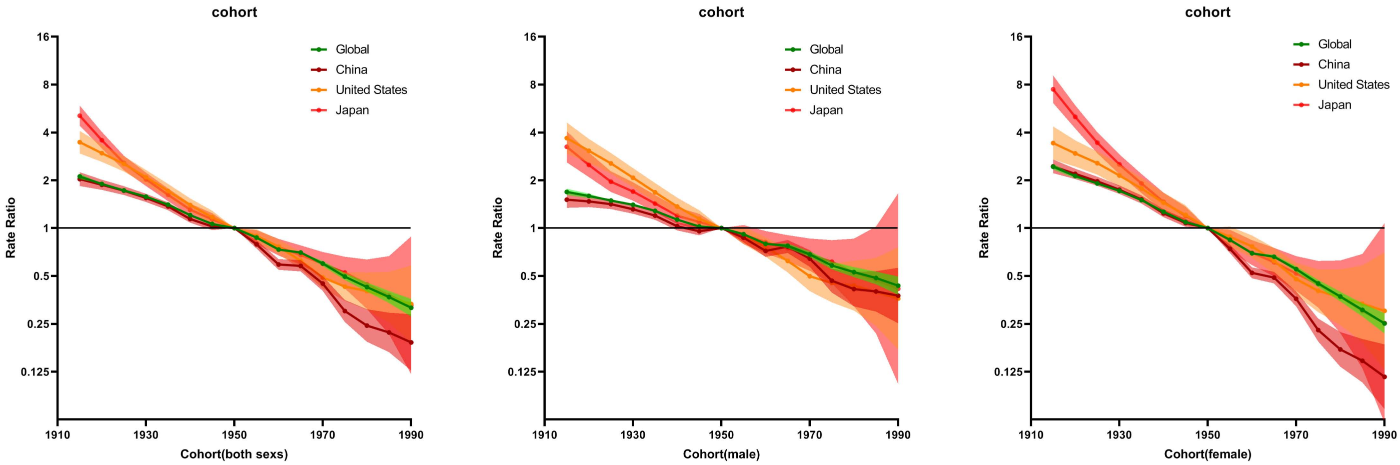

Figure S11(B). The estimated cohort RRs of the mortality rate of stroke attributable to secondhand smoke in global, China, United States and Japan.

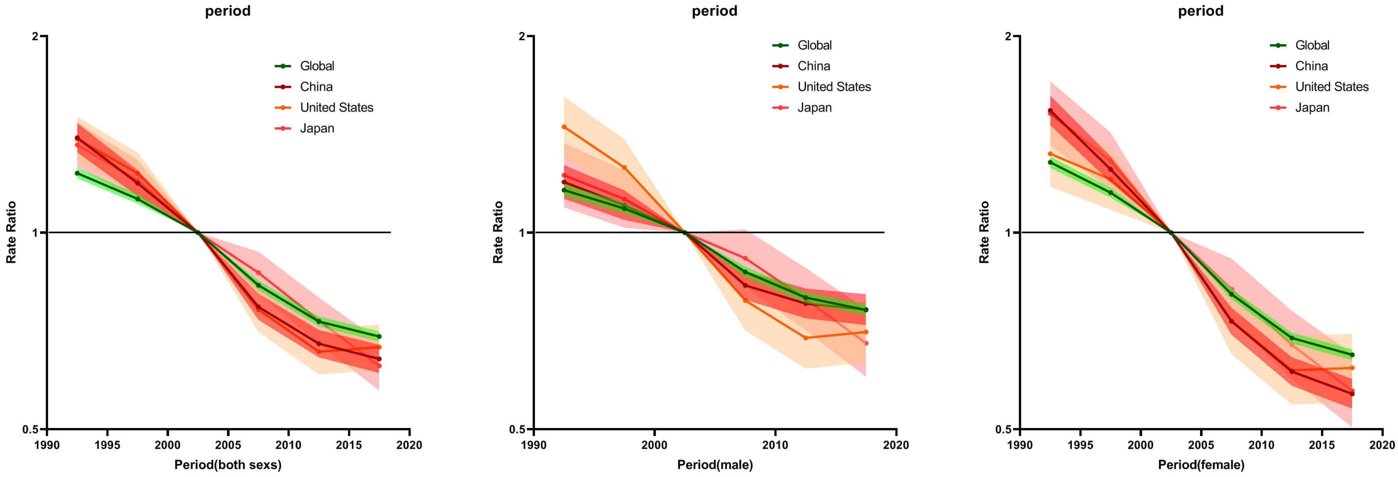

Figure S11(C). The estimated period RRs of the mortality rate of stroke attributable to secondhand smoke in global, China, United States and Japan.

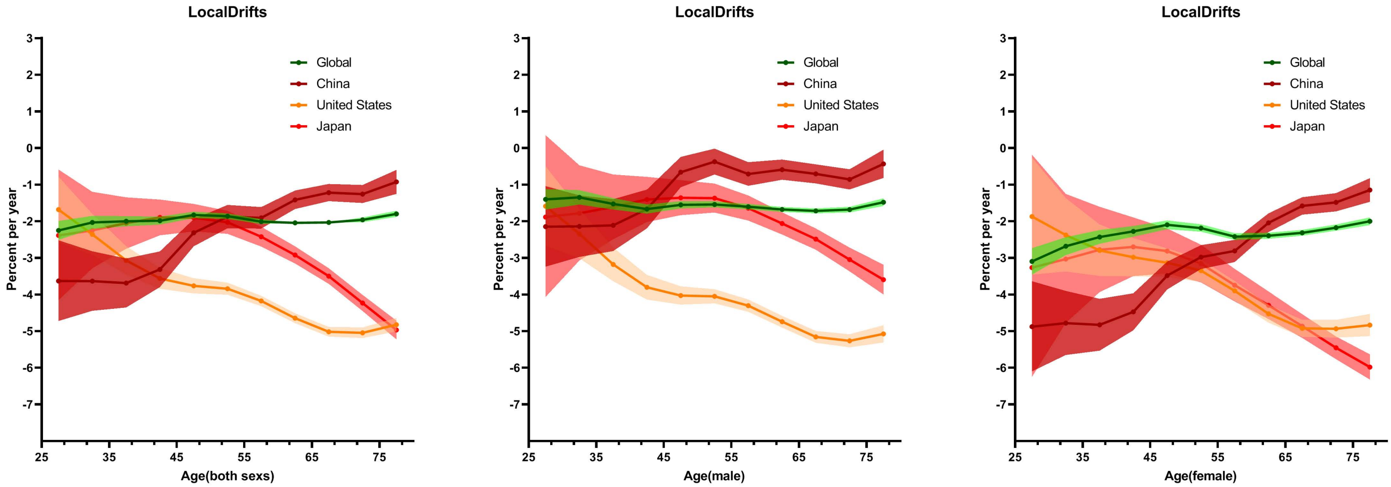

Figure S12(A). The local drift with net drift values of the mortality rate of CVD attributable to secondhand smoke.

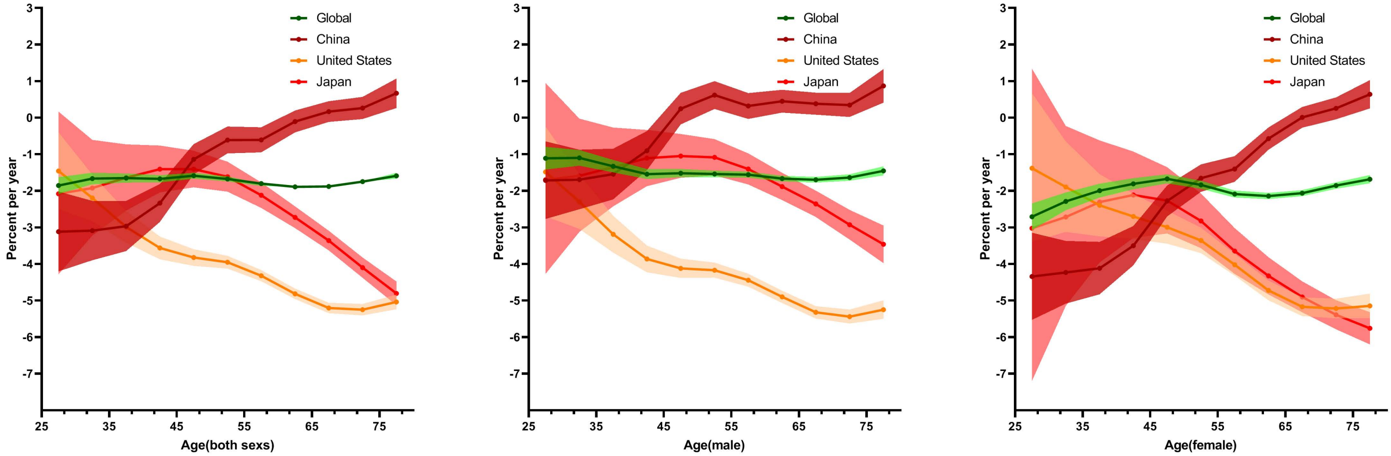

Figure S12(B). The local drift with net drift values of the mortality rate of IHD attributable to secondhand smoke.

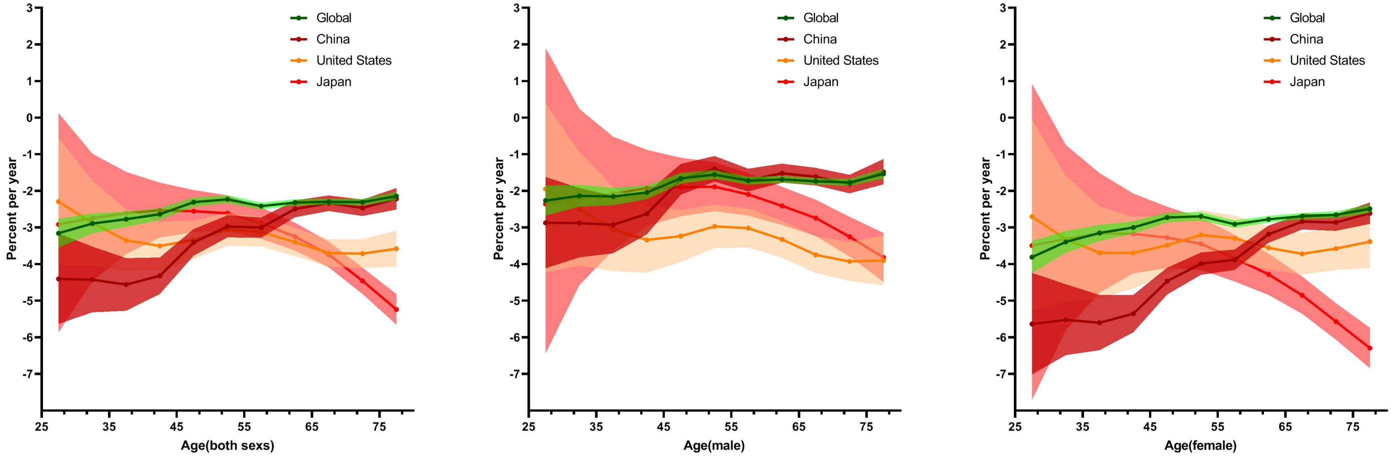

Figure S12(C). The local drift with net drift values of the mortality rate of stroke attributable to secondhand smoke.
